# Similar patterns of genetic diversity and linkage disequilibrium in Western chimpanzees (*Pan troglodytes verus*) and humans indicate highly conserved mechanisms of MHC molecular evolution

**DOI:** 10.1186/s12862-020-01669-6

**Published:** 2020-09-15

**Authors:** Christelle Vangenot, José Manuel Nunes, Gaby M. Doxiadis, Estella S. Poloni, Ronald E. Bontrop, Natasja G. de Groot, Alicia Sanchez-Mazas

**Affiliations:** 1 Laboratory of Anthropology, Genetics and Peopling History, Department of Genetics and Evolution, Anthropology Unit, University of Geneva, Geneva, Switzerland; 2 Institute of Genetics and Genomics in Geneva (iGE3), University of Geneva, Geneva, Switzerland; 3 Comparative Genetics and Refinement, Biomedical Primate Research Centre, 2288 GJ Rijswijk, The Netherlands

**Keywords:** *MHC*, *Patr*, *HLA*, Western chimpanzees, Human populations, Nucleotide diversity, Linkage disequilibrium, Selective sweep, Balancing selection, Population bottleneck, Demographic history

## Abstract

**Background:**

Many species are threatened with extinction as their population sizes decrease with changing environments or face novel pathogenic threats. A reduction of genetic diversity at major histocompatibility complex (*MHC*) genes may have dramatic effects on populations’ survival, as these genes play a key role in adaptive immunity. This might be the case for chimpanzees, the *MHC* genes of which reveal signatures of an ancient selective sweep likely due to a viral epidemic that reduced their population size a few million years ago. To better assess how this past event affected *MHC* variation in chimpanzees compared to humans, we analysed several indexes of genetic diversity and linkage disequilibrium across seven *MHC* genes on four cohorts of chimpanzees and we compared them to those estimated at orthologous *HLA* genes in a large set of human populations.

**Results:**

Interestingly, the analyses uncovered similar patterns of both molecular diversity and linkage disequilibrium across the seven *MHC* genes in chimpanzees and humans. Indeed, in both species the greatest allelic richness and heterozygosity were found at loci *A*, *B*, *C* and *DRB1,* the greatest nucleotide diversity at loci *DRB1*, *DQA1* and *DQB1*, and both significant global linkage disequilibrium and the greatest proportions of haplotypes in linkage disequilibrium were observed at pairs *DQA1 ~ DQB1*, *DQA1 ~ DRB1*, *DQB1 ~ DRB1* and *B ~ C*. Our results also showed that, despite some differences among loci, the levels of genetic diversity and linkage disequilibrium observed in contemporary chimpanzees were globally similar to those estimated in small isolated human populations, in contrast to significant differences compared to large populations.

**Conclusions:**

We conclude, first, that highly conserved mechanisms shaped the diversity of orthologous *MHC* genes in chimpanzees and humans. Furthermore, our findings support the hypothesis that an ancient demographic decline affecting the chimpanzee populations – like that ascribed to a viral epidemic – exerted a substantial effect on the molecular diversity of their *MHC* genes, albeit not more pronounced than that experienced by *HLA* genes in human populations that underwent rapid genetic drift during humans’ peopling history. We thus propose a model where chimpanzees’ *MHC* genes regenerated molecular variation through recombination/gene conversion and/or balancing selection after the selective sweep.

## Background

**Fig. 1:**
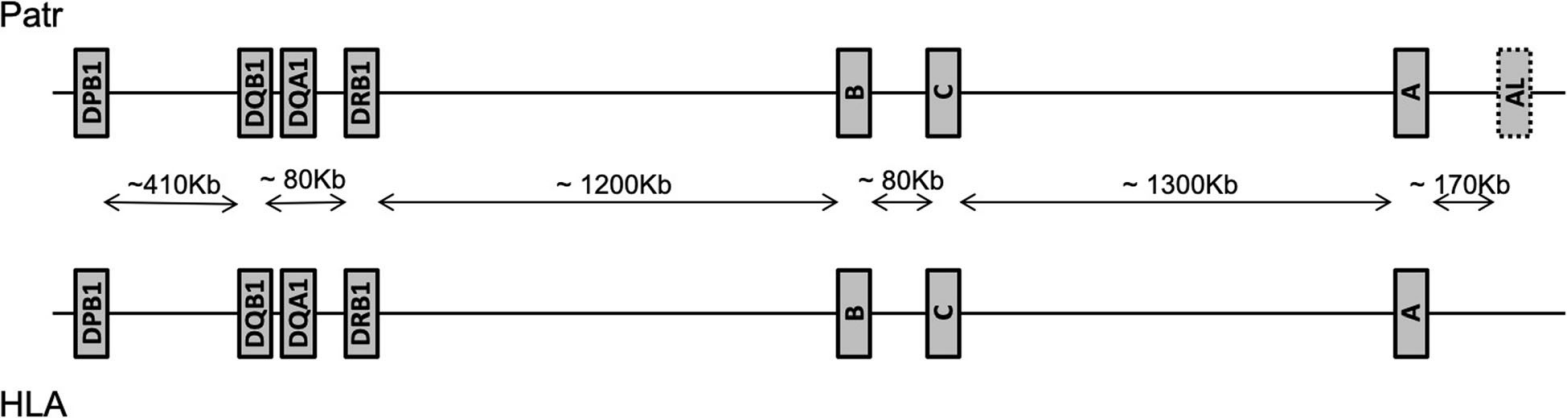
Map of the human and chimpanzee *MHC* region showing average physical distances between the 7 loci under study in both species. The distances between loci (in Kb = kilobases) slightly vary between the two species but they have the same order of magnitude. ~ 80 Kb stands for “physical distance between *DQB1* and *DRB1* is about 80 Kb”

The Major Histocompatibility Complex (*MHC*) is a family of genes that play a major role in activating adaptive immune responses [1]. Some of these gene families code for transmembrane proteins that protect individuals from viral, bacterial and parasitic infections by presenting pathogen-derived peptides to T lymphocytes, which subsequently triggers an immune response. The *MHC* molecular region, called *HLA* in humans and *Patr* in chimpanzees, is very similar in these two species as orthologous genes involved in peptide presentation are physically arranged in a comparable way [2–7] (Fig. 1). These genes are organized into two classes that differ from each other based on major structural and functional differences between their corresponding proteins. The molecules expressed (on almost all nucleated cells) by the classical class I genes (named *A*, *B* and *C*) consist of one α chain, non-covalently bound to a small β2-microglobulin chain which is not encoded in the *MHC* region. The α1 and α2 domains of this heavy chain form the peptide-binding region (PBR) which presents short peptides (mostly nonamers) of intracellular origin at the cell surface to CD8+ cytotoxic T lymphocytes. In all classical *MHC* class I genes, the 2nd and 3rd exons encoding these two domains are highly polymorphic. Chimpanzees may also possess an additional class I *A*-like locus named *Patr-AL* which is in strong linkage disequilibrium with *Patr-A* [8, 9]. However this gene is not fixed but only present on a portion of the haplotypes. The *MHC* molecules encoded by the class II genes (named *DP*, *DQ* and *DR*) display a more specific tissue distribution limited to professional antigen presenting cells implicated in the immune response, i.e. mostly B lymphocytes, dendritic cells and macrophages. Contrary to class I, class II proteins are heterodimers composed of one α chain coded by a “*A*” gene (named *DPA*, *DQA* or *DRA*) and one β chain coded by a “*B*” gene (named *DPB*, *DQB* or *DRB*, respectively). The α1 and β1 domains of the α and β chains form the PBR, which in this case presents peptides (of about 12–15 amino acids) from mostly extracellular origin at the cell surface to CD4+ T-helper lymphocytes. The 2nd exon of most *MHC* class II “*B*” genes (which encodes the β1 domain) is highly variable, whereas that of “*A*” genes (which encodes the α1 domain) is much less polymorphic, except at the *DQ* loci. Most class II genes also exhibit one or more functional and/or non-functional (i.e. pseudogenic) copies (e.g. *DRB1*, *DRB2*, *DRB3*, etc...) resulting from past duplications [5, 10–16], but only the four most polymorphic ones *DPB1*, *DQB1*, *DQA1* and *DRB1* are extensively studied.

The *HLA* region is amongst the most variable of the whole genome, with almost 26,000 *HLA* (class I and class II) alleles identified so far (November 2019, [17]). Its huge level of diversity and/or allelic variation observed within human populations is believed to be maintained by different kinds of balancing selection, most often in the form of heterozygote advantage towards a large variety of pathogens following a divergent allele advantage (DAA) model, although negative frequency-dependent (also named rare-allele advantage) and fluctuating selection in time and space also explain its remarkable variation [18–22]. These mechanisms maintain even *HLA* allele frequencies in most populations, with recurrent – although not systematic – deviations from neutral expectations towards a significant excess of heterozygotes [21, 23]. However, specific *HLA* alleles may also act as protective factors to highly prevalent diseases and be selected positively, one of the best examples being the putative increase of B*53 (B*53:01:01) and B*78 (B*78:01) frequencies in sub-Saharan African regions where *Plasmodium falciparum* malaria is endemic [24–26]. Recently, *MHC* alleles encoding for allotypes with functional similarities to those of *HLA*-B*53 and *HLA*-B*78 have also been suggested to play a protective role explaining the likely absence of malaria parasites in bonobos [27]. In addition, demographic processes such as population bottlenecks, genetic drift, demographic expansions or migrations shape the *HLA* molecular profiles by increasing or decreasing their diversity and create population structure most often highly correlated to geography [21, 28–30].

Whether and how *MHC* genetic variation persists in populations having undergone a pronounced reduction in size, either due to a founder effect or to an epidemic, is an important issue in evolutionary genetics and conservation biology [30–34]. Indeed, a loss of genetic variation, particularly concerning immune-related loci, may have dramatic effects on populations’ survival [33], even though a direct correlation between a lower *MHC* diversity and a greater susceptibility to diseases has not been demonstrated so far at a population level [35, 36]. In this context, theoretical and empirical studies investigating the relative effects of genetic drift and natural selection on *MHC* variability during population bottlenecks in different species have reported contrasting results, indicating either that balancing selection processes were efficient enough to maintain moderate to high *MHC* diversity [31, 37–40] or that demographic factors exerted stronger influence than selection on diversity [41, 42]. Additionally, the impact of selection may depend both on the timescales, e.g. selection would be able to restore diversity to pre-bottleneck levels after 40 generations [31], and on the specific *MHC* gene studied [38, 39, 41].

One useful approach to unravel the multiple mechanisms governing the evolution of the *MHC* region is to compare the diversity of homologous genes among closely related species that underwent distinct demographic histories. This is the case for humans and chimpanzees, which share a common ancestor dating back to ~ 6–8 million years (Myr) ago [43, 44]. According to both archaeological and genetic data, anatomically modern humans (*Homo sapiens*) first appeared and expanded demographically in Africa between 300,000 and 200,000 years ago [45, 46]. They later dispersed, likely in small groups, across all continents where they eventually underwent secondary expansions, the most extensive ones (in Prehistoric times) occurring in the Neolithic [47, 48]. However, many human populations (most Amerindian, Oceanian and present-day hunter-gatherer and nomadic populations from different continents) did not undergo demographic expansions [49] and still live today in isolated areas where they experience little gene flow and rapid genetic drift [50]. Due to the paucity of fossil records [51], the demographic history of chimpanzee populations relies almost exclusively on molecular analyses. The latter suggest the emergence of both common chimpanzees (*Pan troglodytes, P.t.* hereafter) and bonobos (*Pan paniscus*) in Central Africa from a common ancestor ~ 1–2 Myr ago [43, 44]; but while bonobos probably remained confined within the small geographic region where they inhabit today (a narrow territory between the Congo and Kasai Rivers), common chimpanzees expanded across a wider area of equatorial Africa where they are represented today by distinct sub-species (*P.t.verus* in Western Africa, *P.t.ellioti* in Nigeria and Cameroon, *P.t.troglodytes* in Central Africa, and *P.t.schweinfurthii* in Eastern Africa), albeit mainly within a limited rainforest habitat [52–54].

*MHC* molecular data analyses indicated that both common chimpanzees and bonobos experienced a selective sweep owing to the action of a hypothesised retroviral infection that severely shrunk their population sizes (bottleneck events) [55, 56]. The first evidence comes from the observation of a reduced repertoire of allele families at the *Patr-A* locus compared to the *HLA-A* locus in humans [57], suggesting a strong selective sweep – i.e. either purifying or positive directional selection - within the chimpanzees’ *MHC* class I region. Indeed, whereas *HLA-A* alleles belong to six different allele families (*A2*, *A10* and *A19* within the *A2* lineage, and *A1/A3/A11/A30*, *A9* and *A80* within the *A3* lineage), all *Patr-A* alleles known so far are associated to the single *A1/A3/A11/A30* family [57–62] and a similar observation has been reported for the *Papa-A* alleles [63] (*Papa* is the name of *MHC* genes in bonobo). Next, *Patr-* and *Papa-A*, −*B*, −*C* intron 2 analyses substantiated the reduced diversity observed in the Western chimpanzee (*P.t.verus*) and bonobo *MHC* class I regions as compared to *HLA-A*, −*B*, *−C* in humans [55, 63, 64]. In addition, microsatellite analyses in Western chimpanzees and humans revealed a reduced diversity in the *Patr* region in comparison to microsatellites located elsewhere in the genome [56]. Finally, chimpanzees were shown to exhibit a 95 kb deletion in the *MIC* region located next to locus *B* where the single *MIC* gene, which is fixed on all haplotypes, likely results from the fusion of two ancestral *MICA* and *MICB* genes still present in humans [65]. The hypothesis of a selective sweep proposed for chimpanzees finds support in the low genomic diversity found in all common chimpanzee sub-species and in bonobos, which was ascribed to a bottleneck in the ancestors of both species [44]. In addition, these genome-wide analyses also highlighted a second bottleneck occurring later (~ 500,000 years ago) in Western and Nigeria-Cameroon chimpanzees only (although not quite as severe for the latest), which would partially explain why *P.t.verus* generally displays lower molecular variation in nuclear genes compared to other chimpanzee (sub-)species [44, 66–71].

In this study, our objective is to assess whether the genetic diversity at different *Patr* genes, estimated by means of three different indexes, allelic richness, expected heterozygosity and nucleotide diversity*,* is significantly reduced in present-day Western chimpanzee as a possible response to their past bottlenecks compared to that of their *HLA* orthologs in human populations. The detection of a substantially reduced level of *Patr* diversity would be a possible indicator of depleted immunity and an additional reason to consider *P.t.verus* as a critically endangered subspecies [72]. Actually, we anticipate chimpanzees’ *MHC* diversity to be (not necessarily similar but) closer to that of small isolated, as opposed to large outbred human populations (independently of their geographical location) if demographic contractions played a major role on the *MHC* evolution of both species. In addition, we expect the patterns of genetic variation and linkage disequilibrium to be similar across the *HLA* and *Patr* regions if their orthologous loci evolved through analogous molecular mechanisms and were targeted by similar selective pressures in the two species. To address these issues, we analysed all the data currently available for 7 *Patr* genes (*A*, *B*, *C*, *DRB1*, *DQA1*, *DQB1* and *DPB1*) in four *P.t.verus* cohorts, and we compared them to large sets of data for *HLA* genes (*A*, *B*, *C*, *DRB1*, *DQA1*, *DQB1* and *DPB1*) data previously studied in human populations from different continents, that we also extensively reanalysed. We found marked similarities in *Patr* and *HLA* genetic diversity and linkage disequilibrium patterns, indicating highly conserved mechanisms of *MHC* evolution in chimpanzees and humans. We also showed that Western chimpanzees globally exhibit similar diversity levels and equivalent amounts of linkage disequilibrium to those estimated in small isolated human populations, which suggests that their past bottleneck exerted a substantial effect on the molecular diversity of *Patr* genes. However, as there was no difference in the *MHC* diversity of chimpanzees compared to human populations that likely underwent more recent, rapid genetic drift, we hypothesize that several *Patr* genes rapidly recovered molecular variation after their selective sweep.

## Results

### Hardy-Weinberg equilibrium and selective neutrality

The results of Hardy-Weinberg equilibrium (HWE) and Ewens-Watterson-Slatkin (EWS) tests are provided in Table 1 (for the pooled chimpanzee cohort and the multiple human populations) and Additional Tables S1 (for the individual chimpanzee cohorts) and S2 (for the individual human populations).

**Table 1:** Results of Hardy-Weinberg equilibrium (HWE) and Ewens-Watterson-Slatkin (EWS) tests at seven MHC loci in chimpanzees (pooled cohort) and humans (multiple populations)

Chimpanzees (pooled cohort)	*DPB1*	*DQB1*	*DQA1*	*DRB1*	*B*	*C*	*A*
*N*	44	48	29	46	51	51	50
HWE *p-value*	1	0.456	0.627	0.81	0.983	0.226	1
EWS *p-value* (excess heterozygotes)	0.079	0.873	0.100	0.031	0.645	0.811	0.069
EWS *p-value* (excess homozygotes)	0.491	0.970	0.707	0.473	0.907	0.945	0.572
Humans (multiple populations)	*DPB1*	*DQB1*	*DQA1*	*DRB1*	*B*	*C*	*A*
*k*	50	79	52	89	80	59	81
$\overline{N}$*(s.d)*	87.8 (45.2)	100.6 (55.3)	90.7 (45.5)	105.5 (111.8)	124 (134.6)	127.9 (141.1)	127.6 (133.6)
% HWE rejections (after correction)	0	0	0	1.1 (0)	0	0	0
% EWS rejections (excess heterozygotes)	0	16.46 (2.5)	28.84 (0)	30.34 (7.9)	30 (0)	33.9 (0)	16.05 (3.7)
% EWS rejections (excess homozygotes)	22 (2)	1.27 (0)	0	2.25 (0)	5 (0)	1.69 (0)	7.41 (0)

N: sample size (in number of individuals); k: number of populations; $\overline{N}$: mean sample size (in number of individuals); s.d.: standard deviation; Holm correction for multiple testing is given within brackets for the % of HWE and EWS tests. Significance tests were done without prior assumptions, thus two-tailed rejection at the 5% level either occurs below 0.025 (excess of heterozygotes) or above 0.975 (excess of homozygotes). The order of loci corresponds to their position on the chromosome from centromere (left) to telomere (right). The pooled cohort does not include Texas^cb^ (see Text)

No deviation from HWE was observed at any *Patr* locus for any of the four individual cohorts and the pooled cohort of chimpanzees. The computed allele frequencies (see below) could thus accurately be used as population frequencies to compare cohorts among them and with human populations as well as to estimate other parameters requiring HWE (e.g. heterozygosity). Additionally, we found no significant deviations (after correction for multiple testing) of allele frequency distributions from neutral expectations based on the EWS test.

All human populations were also found to be in HWE both before (except the Mixe (Mexico/Oaxaca) at DRB1) and after correction for multiple testing. Contrary to chimpanzees, however, a few significant rejections of selective neutrality were still found in human populations after correction for multiple testing, i.e. towards an excess of heterozygotes at loci *A* (3.7%), *DRB1* (7.9%) and *DQB1* (2.5%) and towards an excess of homozygotes at locus *DPB1* (2%), but none at loci *DQA1*, *B* and *C*.

To control for the large differences in sample sizes between chimpanzees (average *N* = 45.57 ± 7.76 on the 7 loci in the pooled cohort) and humans (average *N* = 109.2 ± 17.31 on the 7 loci and the multiple populations), we also tested HWE and selective neutrality on 1000 simulated sub-samples drawn randomly from each human population, each simulated sub-sample being of same size as the pooled cohort of chimpanzees (see Methods). As a result, we observed various proportions of HWE deviations in the simulated sub-samples depending on the locus (average proportion ± 2xStandard Error, *DPB1*: 8.05% ± 8.20%, *DQB1*: 13.38 ± 9.83%, *DQA1*: 6.89 ± 9.40%, *DRB1*: 10.37 ± 8.99%, *B*: 3.36 ± 5.05%, *C*: 3.20 ± 4.93%, *A*: 5.77 ± 6.6%, Additional Table S2**)**. As almost all human populations of the original dataset were in HWE, this overall result allowed us to conclude that a reduction in sample size sometimes leads to type I errors, i.e. false positives, at loci *DQB1* and *DRB1* (the only proportions significantly different from 0). However, for the Mixe from Mexico/Oaxaca, which was the only population for which HWE was rejected before correction for multiple testing (at locus *DRB1*) in the original dataset, HWE was rejected in all (i.e. the 1000) simulated sub-samples, a result that never occurred otherwise (Additional Table S2). This indicates that the power of the test strongly resists a reduction in sample size, and that the observation of no HWE rejection in the chimpanzee samples truly reflects HWE in the corresponding cohorts.

Regarding selective neutrality, our simulations failed to reject the null hypothesis in various proportions of simulated sub-samples drawn from populations for which neutrality was initially rejected (10% at locus *DPB1*, 2.1% at locus *DQB1*, 28.3% at locus *DRB1* and 18.4% at locus *A*, Additional Table S2). In this case, the absence of significant deviations from neutrality observed in chimpanzees could thus correspond to type II errors, i.e. false negatives, due to a lack of power of the neutrality test when applied to small sample sizes, although this occurred in a minority of cases according to our simulations (less than 30%).

### Genetic diversity

Allele frequencies estimated in the pooled cohort of chimpanzees are given in Table 2 and Additional Table S1 (for the individual chimpanzee cohorts). Allelic distributions found at the three class I loci *B, C* and *A* and at *DRB1* are much more diverse than those observed at *DQB1* and *DQA1* and, to a lesser extent, *DPB1*. Moreover, at loci *DQB1* and *DQA1*, three alleles account for more than 84.5% of frequencies. A greater number of low frequency alleles are observed for loci *B, C* and *A* than for class II loci (in light grey in Table 2, see also SupplementaryText for a comparison between chimpanzee cohorts and human populations).

**Table 2:** Allele frequencies at each *Patr* locus in the pooled cohort of chimpanzees^a^

Locus *DPB1*		Locus *DQB1*		Locus *DQA1*^b^		Locus *DRB1*		Locus *B*		Locus *C*		Locus *A*	
*N* = 44		*N* = 48		*N* = 29		*N* = 46		*N* = 51		*N* = 51		*N* = 50	
DPB1*01:09	0.279	DQB1*03:02	0.5937	DQA1*20:04	0.4138	DRB1*02:01	0.2283	B*01:01	0.2807	C*04:01	0.3075	A*03:01	0.1505
DPB1*01:11	0.2514	DQB1*06:02	0.2917	DQA1*01:01	0.2759	DRB1*02:04	0.2283	B*05:01	0.1764	C*06:01	0.2629	A*04:01	0.126
DPB1*01:12	0.1705	DQB1*06:01	0.0521	DQA1*05:02	0.1552	DRB1*03:07	0.1528	B*13:01	0.0784	C*09:01	0.1211	A*09:01	0.126
DPB1*01:07	0.1073	DQB1*15:01	0.0312	DQA1*20:01	0.1034	DRB1*03:02	0.0913	B*24:02	0.0783	C*02:03	0.0686	A*07:01	0.1193
DPB1*01:13	0.1023	DQB1*03:05	0.0208	DQA1*05:03	0.0517	DRB1*03:05	0.087	B*17:01	0.0686	C*11:01	0.049	A*01:01	0.0983
DPB1*01:18	0.0114	DQB1*06:07	0.0104			DRB1*03:09	0.0462	B*14:01	0.0588	C*05:01	0.0392	A*14:01	0.07
DPB1*03:04	0.0114					DRB1*10:01	0.0435	B*24:01	0.0392	C*12:01	0.033	A*06:01	0.0668
blank	0.0668					DRB1*03:11	0.0326	B*04:01	0.0294	C*03:01	0.0196	A*05:01	0.0422
						DRB1*07:01	0.0217	B*16:01	0.0294	C*05:02	0.0196	A*02:01	0.0319
						blank	0.0684	B*20:01	0.0294	C*01:01	0.0114	A*11:01	0.03
								B*03:01	0.0214	C*02:02	0.0098	A*06:02	0.0106
								B*09:01	0.0214	C*08:01	0.0098	A*03:02	0.01
								B*29:01	0.0196	C*09:02	0.0098	A*04:02	0.01
								B*05:02	0.0098	C*13:02	0.0098	A*04:04	0.01
								B*08:02	0.0098	blank	0.0288	A*08:02	0.01
								B*10:01	0.0098			A*08:03	0.01
								B*16:02	0.0098			A*17:01	0.01
								B*19:01	0.0098			blank	0.0683
								B*22:01	0.0098				
								B*23:01	0.0098				
								blank	0.0002				

Alleles in grey have a frequency lower than 0.05

^a^The allele frequencies for the individual chimpanzee cohorts are in Additional Table S1

^b^For locus *DQA1*, only data for cohort BPRC^wb^ are available

**Fig. 2:**
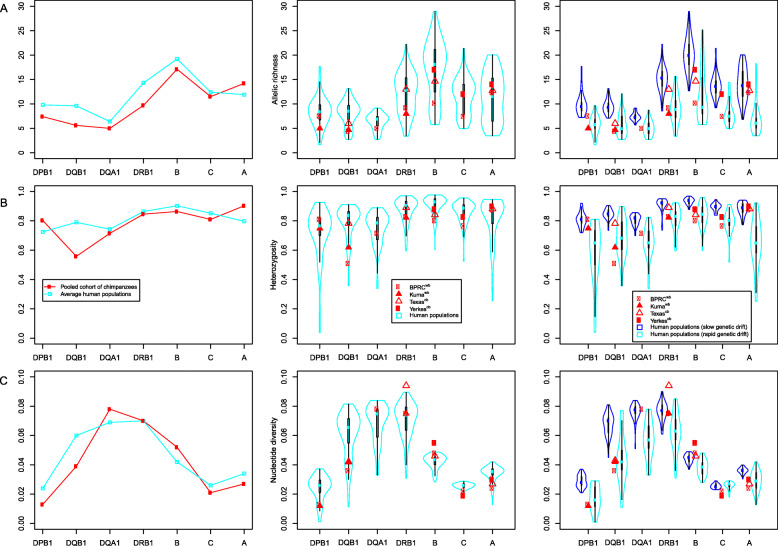
Genetic diversity indexes estimated in chimpanzee cohorts and human populations. Left panels: allelic richness (top), heterozygosity (middle) and nucleotide diversity (bottom) at the seven studied *MHC* loci in the pooled cohort of chimpanzees (in red) and averaged on multiple human populations (in blue). The pooled cohort includes all cohorts except Texas^cb^. Middle panels: allelic richness (top), heterozygosity (middle) and nucleotide diversity (bottom) at the seven studied *MHC* loci in each cohort of chimpanzees (in red) and for the human populations (in blue) represented as violin plots. The values calculated for each chimpanzee cohort are indicated by filled and unfilled shapes for cohorts of wild-born and captive-born chimpanzees, respectively. The values calculated for the human populations (average number of k = 70 (s.d 15.9) samples of average size *N* = 109.2 (s.d 17.31)) are shown as violin plots. The width of the violin varies so as to represent the probability density of the data, the thick black bar in the centre represents the interquartile range, the thin black line extended from it represents the 95% confidence intervals, and the blue dot is the median. Right panel: allelic richness (top), heterozygosity (middle) and nucleotide diversity (bottom) at the seven studied *MHC* loci in each cohort of chimpanzees (in red) and for the human populations (in two shades of blue) represented as violin plots. The values calculated for each chimpanzee cohort are indicated by filled and unfilled shapes for cohorts of wild-born and captive-born chimpanzees, respectively. The values calculated for the human population are plotted as violin plots, in light blue for small sized and isolated populations that likely experienced rapid genetic drift (RGD) and in dark blue for large outbred populations with slow genetic drift (SGD).

The three genetic diversity indexes estimated at the seven *Patr* genes in the four cohorts and the pooled cohort of chimpanzees are given in Table 3 and plotted in Fig. 2, and the corresponding values are provided in Additional Table S3.

**Table 3:** Genetic diversity at 7 MHC loci in chimpanzees (average on all chimpanzee cohorts and in the pooled cohort) and human populations (averaged on multiple populations)

	Number of samples (*k*) & sample size (*N*)				Allelic richness (*ar*)			Heterozygosity (*H*)			Nucleotide diversity (*П*)		
	Chimpanzees		Humans		Chimpanzees		Humans	Chimpanzees		Humans	Chimpanzees		Humans
					average	pooled^a^		average	pooled^a^		average	pooled^a^	
	*k*	*N*^*P*^	*k*	$\overline{N}$*(s.d)*	$\overline{ar\ }$*(s.d)*	*ar*^*P*^	$\overline{ar}$^*P*^ *(s.d)*	$\overline{H}\%$*(s.d)*	*H*^*P*^*%*	$\overline{H}\%$*(s.d)*	$\overline{\varPi}$*x* 10^-2^ *(s.d)*	*П*^*P*^ x 10^-2^	$\overline{\varPi}$*x* 10^-2^ *(s.d)*
*DPB1*	2	44	50	87.8 *(45.2)*	6.3 *(1.8)*	7.4	9.8 *(3.7)*	77.9 *(0.4)*	80.3	72.5 *(17.1)*	1.3 *(0.1)*	1.3	2.4 *(0.9)*
*DQB1*	3	48	79	100.6 *(55.3)*	5.0* *(0.9)*	5.6	9.6 *(3.2)*	63.7* *(13.8)*	55.8	79 *(11.2)*	4.0* *(0.3)*	3.9	6.0 *(1.6)*
*DQA1*	1	29	52	90.6 *(45.5)*	5 *(-)*	5	6.4 *(1.6)*	71.5 *(-)*	71.5	74.3 *(10.9)*	7.8 *(-)*	7.8	6.9 *(1.3)*
*DRB1*	3	46	89	105.5 *(111.8)*	10.1 *(2.6)*	9.7	14.3 *(5.3)*	84.7 *(3.8)*	84.6	86.4 *(8.3)*	8.1 *(1.1)*	7.0	7.0 *(1.2)*
*B*	3	51	80	124 *(134.6)*	13.9 *(3.5)*	17.1	19.2 *(7.1)*	84.1 *(3.9)*	86.4	90.3 *(6.8)*	5.0* *(0.5)*	5.2	4.2 *(0.5)*
*C*	2	51	59	127.9 *(141.1)*	9.7 *(3.2)*	11.5	12.4 *(4.2)*	79.6 *(4.3)*	81	85.2 *(7.4)*	2.1* *(0.2)*	2.1	2.6 *(0.2)*
*A*	3	50	81	127.6 *(133.6)*	13 *(0.9)*	14.2	11.9 *(5.2)*	88.6 *(1.2)*	90.3	79.7 *(15.4)*	2.7* *(0.3)*	2.7	3.4 *(0.5)*

*k*: number of samples; *N*^*p*^: size of pooled cohort; $\overline{N}$*(s.d)*: average size of human population samples and standard deviation

$\overline{ar}$*(s.d):* average allelic richness in the chimpanzee samples and standard deviation; *ar*^*P*^: allelic richness of the pooled cohort; $\overline{ar}$^*P*^ *(s.d):* average allelic richness in human population samples (estimated relatively to the pooled cohort) and standard deviation. * value (in bold) significantly different between human populations and chimpanzee cohorts (Wilcoxon test, *p* = 0.0342) for allelic richness at *DQB1*. Test without the Texas^cb^ cohort: Wilcoxon two-sided test: *p* = 0.0604, single-sided test (“less”): *p* = 0.0302

$\overline{H}\%$*(s.d):* average expected heterozygosity in the chimpanzee samples and in human population samples and standard deviation; *H*^*P*^*%*: expected heterozygosity of the pooled cohort. * value (in bold) significantly different between human populations and chimpanzee cohorts (Wilcoxon test, *p* = 0.0316) for heterozygosity at *DQB1*. Test without the Texas^cb^ cohort: Wilcoxon two-sided test: *p* = 0.0371, single-sided test (“less”): *p* = 0.0185

$\overline{\varPi}$*(s.d):* average expected nucleotide diversity in the chimpanzee samples and in human population samples and standard deviation; *П*^*P*^: expected nucleotide diversity of the pooled cohort. * values (in bold) significantly different between human populations and chimpanzee cohorts (Wilcoxon test, *p* = 0.0426, 0.0175, 0.0225,0.0371) for nucleotide diversity at *DQB1*, *B*, *C* and *A*, respectively. Test without the Texas^cb^ cohort: Wilcoxon two-sided test: *p* = 0.0825, 0.0255, 0.0944, single-sided test: *p* = 0.0412, 0.0128, 0.0472 at *DQB1*, *B* and *A*, respectively

The order of loci corresponds to their position on the chromosome from centromere (top) to telomere (bottom)

^a^does not include Texas^cb^ (see Text)

In agreement with the observed allele frequency distributions, both allelic richness and heterozygosity show greater values at the three class I loci *A*, *B*, *C* and at *DRB1* than at *DQA1*, *DQB1* and *DPB1* (to a lesser extent for the latter). Based on the loci for which data were available in (at least one) captive and wild cohorts (*DQB1, DRB1, B, C, A*), we also observe significantly higher values of these indexes in the captive-born Texas^cb^ and Yerkes^cb^ than in the wild-born BPRC^wb^ and Kuma^wb^ cohorts (Wilcoxon tests, *p* = 0.0036 and *p* = 0.0034, for allelic richness and heterozygosity, respectively). By contrast, nucleotide diversity is greater at *DRB1, DQA1*, *DQB1* and *B* (to a lesser extent in the two latter) than at *A*, *C* and *DPB1*, and no significant differences are observed between the cohorts (Wilcoxon test, *p* = 0.769).

Like in chimpanzees, both the allelic richness and the heterozygosity estimated in human populations are, on average, greater at the three class I loci *A*, *B*, *C* and at *DRB1* than at *DQA1*, *DQB1* and *DPB1* and the nucleotide diversity is greater at loci *DRB1*, *DQA1, DQB1* and *B* than at *A*, *C* and *DPB1* (Table 3 and Fig. 2). The overall patterns of genetic diversity are therefore similar in the two species. This is also supported by comparing the ordering of the seven *MHC* loci based on decreasing values of the three diversity indexes (Table 4): identical orders are found for several loci, and small differences are most often observed otherwise. These results suggest that the mechanisms generating diversity at the *MHC* genes are similar, and thus highly conserved, in the human and chimpanzee lineages.

**Table 4:** Ordering of the MHC loci based on decreasing values of three genetic diversity indexes in chimpanzees (pooled cohort) and humans (average on multiple populations)

Genetic diversity	Species	Ordering of values
Allelic richness	Chimpanzees (pooled cohort^&^)	***B*** *> A >* ***C*** *> DRB1 >* ***DPB1 > DQB1 > DQA1***
	Humans (average^#^)	***B*** *> DRB1 >* ***C*** *> A >* ***DPB1 > DQB1 > DQA1***
Heterozygosity	Chimpanzees (pooled cohort^&^)	*A >* ***B > DRB1 > C*** *> DPB1 >* ***DQA1 >*** *DQB1*
	Humans (average^#^)	***B > DRB1 > C*** *> A > DQB1 >* ***DQA1*** *> DPB1*
Nucleotide diversity	Chimpanzees (pooled cohort^&^)	*DQA1 >* ***DRB1*** *> B >* ***DQB1*** *>* ***A > C > DPB1***
	Humans (average^#^)	***DRB1*** *> DQA1 >* ***DQB1*** *> B >* ***A > C > DPB1***

Perfect matches in locus ordering between chimpanzees and humans are highlighted in bold; ^#^ estimated on multiple human populations; ^&^: for chimpanzees, the pooled cohort has an average sample size of 45.57 on the different loci; ^#^: for humans, the averages were calculated on an average number of 70.14 populations with an average sample size of 109.46. The pooled cohort does not include Texas^cb^ (see Text)

Looking in more detail at the results obtained for individual *MHC* genes, some significant differences are nevertheless observed between the two species. Compared to humans, in chimpanzees we find a lower heterozygosity, allelic richness and nucleotide diversity at *DQB1* (Wilcoxon test, *p* = 0.016, 0.017 and 0.021, respectively), as well as a lower nucleotide diversity at *C* and *A* (Wilcoxon test, *p* = 0.011 and 0.019, respectively) and a higher nucleotide diversity at *B* (Wilcoxon test, *p* = 0.009) (Table 3 and Fig. 2). We obtained similar results by redoing these comparisons without considering the Texas^cb^ cohort, which includes individuals of uncertain sub-species (Wilcoxon test: *p* = 0.019, 0.03 and 0.041 for heterozygosity, allelic richness and nucleotide diversity at *DQB1*; and *p* = 0.025, 0.013 and 0.047 for nucleotide diversity at *B*, *C*, and *A,* respectively, see also Additional Figure S1). However, according to both sets of comparisons (i.e. with and without the Texas^cb^ cohort), none of these differences remained significant after correction for multiple testing on the number of loci. This confirmed our previous conclusion that chimpanzees and humans display similar patterns of genetic diversity across the whole *MHC* region (Fig. 2, left and central panes).

### Genetic diversity in chimpanzees compared to small and large human populations

Following the idea, based on demographic knowledge, that chimpanzees would be genetically more similar to human populations displaying limited population sizes, we also compared the three diversity indexes between the chimpanzees and the human populations classified either as *RGD* (small isolated populations that likely underwent Rapid Genetic Drift) or as *SGD* (large outbred populations those that likely underwent Slow Genetic Drift), respectively (see Methods).

Interestingly, in the chimpanzee cohorts - and particularly so in the wild-born BPRC^wb^ and Kuma^wb^ - both the allelic richness and heterozygosity (at all loci except *A*) are close to the lowest values found for these indexes in human populations, which correspond to those observed in *RGD* populations (Fig. 2, right graphs). Actually, at these loci, chimpanzees exhibit no significant differences compared to *RGD* populations, whereas all differences (except heterozygosity at *DPB1*) are significant compared to *SGD* populations. In addition, chimpanzees exhibit significant nucleotide diversity differences compared to *SGD* populations at three loci, *DPB1*, *DQB1* and *A* (Additional Table S4). After correction for the number of loci tested, the three diversity indexes appear to be both similar between chimpanzees and *RGD* populations at all loci (except one borderline case, nucleotide diversity at locus *B*) and different between chimpanzees and *SGD* populations (at least two loci remain highly significant after correction). This strongly suggests that demographic contractions globally exerted a similar effect – i.e. a decrease in the level of diversity - on *Patr* and *HLA* genes.

Again to control for the discrepancy in sample sizes between chimpanzees and humans, we re-estimated allelic richness, heterozygosity and nucleotide diversity on 1000 simulated sub-samples randomly drawn for each human population. For the three diversity indexes, the values (in all cases at a precision of one decimal, but most often, even at two) observed for the original human population samples were always found to fall within the 95% confidence interval of their simulated sub-samples (Additional Table S2). In addition, the relative position of each genetic diversity index observed in the pooled cohort of chimpanzees - i.e. either within or outside the 95% confidence interval - was identical when compared both to the confidence interval of the original human population samples and to that of the 1000 simulated sub-samples (Additional Figure S2). This substantiated our previous conclusion that chimpanzees and human *RGD* populations exhibit similar *MHC* diversity patterns.

### Linkage disequilibrium

In chimpanzees, global linkage disequilibrium (GLD) appears to be significant between the three class II loci *DQA1*, *DQB1* and *DRB1* (i.e. pairs *DQA1* ~ *DRB1*, *DQB1* ~ *DRB1* and *DQB1* ~ *DQA1*) as well as between the two class I loci *B* and *C* (pair *B* ~ *C*) (Table 5, see also SupplementaryText), as indicated by the results obtained for the BPRC^wb^ cohort, i.e. the cohort including the greatest number of animals and the only one for which all loci were tested (Additional Table S5). These pairs of loci (actually those that are most close to each other on the chromosome, see Fig. 1) also display the highest proportions of individual haplotypes in linkage disequilibrium (Additional Tables S6 and S7), which strongly supports the observed GLD pattern.

**Table 5:** Results of Global Linkage Disequilibrium (GLD) significance test (PRS resampling procedure) between different pairs of MHC loci in chimpanzees (BPRC cohort) and humans (multiple populations, further subdivided into RGD and SGD populations)

	Chimpanzees		Humans					
	BPRC^WB^		Multiple populations		RGD		SGD	
Loci pairs	*N*	*p*	*k*	*% significant results (after correction)*	*k1*	*% significant results (after correction)*	*k2*	*% significant results (after correction)*
*DPB1 ~ DQB1*	25	0.106	40	30 (12.5)	15	47 (27)	25	24 (4)
*DPB1 ~ DQA1*	25	0.0697	33	27.3 (15.1)	16	44 (25)	17	12 (5.9)
*DPB1 ~ DRB1*	25	0.328	31	25.8 (9.7)	16	37 (12.5)	15	13 (6.7)
*DPB1 ~ B*	25	1	10	10 (10)	4	25 (25)	6	0
*DPB1 ~ C*	25	0.999	8	12.5 (0)	2	50 (0)	6	0
*DPB1 ~ A*	24	0.982	12	0	5	0	7	0
*DQB1 ~ DQA1*	29	**< 10**^**−4**^	46	**97.8 (97.8)**	20	**95 (95)**	26	**100 (100)**
*DQB1 ~ DRB1*	29	**< 10**^**−4**^	51	**94.1 (94.1)**	23	**96 (96)**	28	**93 (93)**
*DQB1 ~ B*	29	1	13	61.5 (53.8)	7	86 (86)	6	33 (16.7)
*DQB1 ~ C*	29	0.975	10	40 (40)	5	60 (60)	5	20 (20)
*DQB1 ~ A*	28	0.98	16	12.5 (12.5)	9	22 (22)	7	0
*DQA1 ~ DRB1*	29	**< 10**^**−4**^	38	**97.4 (97.4)**	21	**100 (100)**	17	**94 (94)**
*DQA1 ~ B*	29	1	9	44.4 (44.4)	5	80 (80)	4	0
*DQA1 ~ C*	29	1	6	33.3 (33.3)	3	67 (67)	3	0
*DQA1 ~ A*	28	0.831	12	8.3 (0)	7	14 (0)	5	0
*DRB1 ~ B*	29	1	39	51.3 (35.9)	22	73 (54.5)	17	23 (11.8)
*DRB1 ~ C*	29	0.998	30	63.3 (63.3)	18	78 (78)	12	42 (42)
*DRB1 ~ A*	28	0.991	39	28.2 (25.6)	24	33 (33)	15	20 (13.3)
*B ~ C*	29	**0.0001**	59	**74.6 (74.6)**	21	**95 (95)**	38	63 (63)
*B ~ A*	28	0.931	76	35.5 (25)	27	63 (40.7)	49	20 (16.3)
*C ~ A*	28	0.892	58	53.4 (37.9)	23	65 (47.8)	35	46 (31.4)

The PRS resampling procedure was done with 10′000 simulations for the chimpanzee cohorts (significant results are in bold). The PRS resampling procedure was done with 1′000 simulations in each human population, and the table reports the percentage of significant results for each pair of loci (in brackets after Holm correction for multiple testing). Pairs in significant global linkage disequilibrium in more than 70% of human populations are in bold. *N*: number of chimpanzee individuals; *p*: *p*-value; *k*: total number of human populations; *k1*: number of *RGD* populations; *k2*: number of *SGD* populations. The table includes results obtained for the BPRC^wb^ cohort, i.e. the cohort including the greatest number of animals and the only one for which all loci were tested. Results for the other cohorts are in Additional Table S5

These results are again similar in humans. Indeed, significant GLD is observed for the same pairs of loci *DQA1* ~ *DRB1*, *DQB1* ~ *DRB1*, *DQB1* ~ *DQA1* and *B* ~ *C* in the majority (more than 70% and up to 98%) of human populations (Table 5**)**, and the highest proportions of individual haplotypes in significant linkage disequilibrium are also observed at these loci pairs in humans (Additional Table S6 and Additional Table S8, respectively). Therefore, as for genetic diversity, the patterns of linkage disequilibrium observed across the *MHC* loci are highly conserved in the human and chimpanzee lineages.

### Linkage disequilibrium in chimpanzees compared to small and large human populations

When comparing human *RGD* and *SGD* populations, the highest proportion of significant GLD are always found among the former, except for one pair of loci, *DQB1 ~ DQA1* (Table 5). Actually, we find both significantly higher proportions of GLD and significantly higher average proportions of haplotypes in linkage disequilibrium in *RGD* than in *SGD* populations (Wilcoxon test: *p* = 0.014 and *p* = 0.012) (Additional Table S6 and Additional Table S8), which indicates that, globally, demography (i.e. genetic drift) did play a substantial role in the generation of linkage disequilibrium at the *HLA* loci. However, this effect appears to be less pronounced at the *DQA1* ~ *DRB1*, *DQB1* ~ *DRB1*, *DQB1* ~ *DQA1* pairs.

Simulations performed on 1000 randomly drawn human population sub-samples show a tendency to under-estimate GLD when sample sizes are low except for pairs *DQB1 ~ DQA1, DQB1 ~ DRB1, DQA1 ~ DRB1* and *B ~ C* (considering samples with GLD in more than 900 sub-samples, we observe between half to two thirds less GLD in the simulated sub-samples except at these four loci pairs) (Additional Table S9). This suggests that the non-detection of significant GLD in chimpanzees for other loci than *DQA1* ~ *DRB1*, *DQB1* ~ *DRB1*, *DQB1* ~ *DQA1* and *B* ~ *C* has a substantial probability to be due to type II errors (false negatives). Regarding individual haplotypes, the proportion of haplotypes in significant LD among 1000 simulated sub-samples drawn from human populations is largely under-estimated, being on average 1.5 to 2 times lower than in the original samples (Additional Table S9). Again this suggests that the proportion of individual haplotypes in significant LD is mostly underestimated in chimpanzees, which may explain why it is up to 3 times lower than that observed in humans at most pairs of loci (Additional Table S6**)**. This means that, overall, chimpanzees are expected to display more GLD and more haplotypes in significant LD than observed in our study, which supports our previous conclusion of their greater resemblance to *RGD* than to *SGD* populations.

## Discussion

### Strong conservation of MHC diversity patterns in humans and chimpanzees

Based on three distinct and complementary statistics describing genetic variation within populations - allelic richness, heterozygosity and nucleotide diversity -, this study has disclosed highly similar patterns of genetic diversity across seven orthologous *MHC* loci in chimpanzees and humans: overall, both allelic richness and heterozygosity are greater at the three class I loci *A*, *B*, *C* and at *DRB1* than at *DQA1*, *DQB1* and *DPB1,* and nucleotide diversity is greater at loci *DRB1*, *DQA1, DQB1* and *B* than at *A*, *C* and *DPB1* (Fig. 2 and Table 4**)**. In addition, based on both global tests and individual haplotypes’ counting, we found similar patterns of linkage disequilibrium across *Patr* and *HLA* genes: both highly significant GLD and the highest proportions of individual haplotypes in significant linkage disequilibrium are observed for the same pairs of loci *DQA1* ~ *DRB1*, *DQB1* ~ *DRB1*, *DQB1* ~ *DQA1* and *B* ~ *C* (Table 5 and Additional Table S6**)**, which parallels the strong resemblance between *Patr* and *HLA* physical maps in chimpanzees and humans, respectively (Fig. 1). These results indicate that the *MHC* diversity patterns are highly conserved in the human and chimpanzee lineages and that analogous mechanisms drove the evolution of this genomic region in the two species since their divergence from a common ancestor.

### Molecular mechanisms generating diversity at MHC genes

In support to the hypothesis that analogous mechanisms drove the evolution of the *MHC* region in chimpanzees and humans, it has been suggested that the molecular processes generating nucleotide (and hence also allelic) diversity at most *MHC* loci are similar in both species: new variants would be mainly generated through point mutations at loci *DQB1*, *DQA1*, *C* and *A,* through recombination and/or gene conversion at loci *DRB1* and *B*, and through both kinds of mechanisms at *DPB1* [58, 73]. This would partly explain why loci *DRB1* and *B* most often exhibit higher nucleotide and allelic diversity than the other class I and class II loci. Interestingly, chimpanzees contrast with macaques [74, 75] and (to some extent) orangutans [76] and gorillas [77], as the *MHC* polymorphism of these species (*Mamu*, *Popy* and *Gogo*, respectively) would also evolve through gene duplications at both loci *B* and *A*.

### Signatures of demography on *Patr* and *HLA* loci

Besides the mechanisms generating diversity at the molecular level, both demographic processes and natural selection are known to shape the patterns of populations’ genetic diversity at *MHC* genes, with possible confounding effects [30, 31, 78]. In this regard, it has been suggested that chimpanzees and humans underwent distinct demographic histories [44, 54, 66, 69, 71, 79–83] that probably affected in different ways their *MHC* profiles [55, 56, 61, 62]. However, demographic evolution has not been uniform in all human populations either [49]. In order to better disentangle the evolutionary mechanisms that drove the evolution of *MHC* genes in the two species, we thus compared chimpanzees to many different human populations displaying a wide diversity of demographic histories [84] and living in distinct geographical locations – and hence being also submitted to very diverse environmental pressures [23, 85].

As expected, large ranges of genetic diversity values were observed among human populations (Fig. 2). Interestingly, the three genetic diversity indexes - allelic richness, heterozygosity, and nucleotide diversity - appeared to be similar between chimpanzees (especially the wild-born cohorts BPRC^wb^ and Kuma^wb^) and the small isolated human populations that likely underwent rapid genetic drift (*RGD*), regardless of the geographic regions or continents where these human populations lived, and different between chimpanzees and the large outbred (*SGD*) human populations (Fig. 2 and Additional Figure S3). As an example, the very low nucleotide diversity found at the four *Patr* genes *DPB1*, *DQB1*, *C* and *A* is comparable to that found at the orthologous *HLA* genes in Amerindians and Australian Aborigines (Additional Figure S3**)** as examples of *RGD* populations. Because neither human populations living in America and Australia nor chimpanzees living in sub-Saharan Africa likely experienced the same pathogenic pressures, comparable demographic histories (i.e. limited population sizes) better explain the similarities than convergent selective effects. Regarding linkage disequilibrium, our simulations indicated that we probably underestimated the amount of GLD and individual haplotypes in significant LD in chimpanzees. This plays in favour of a putative greater resemblance between chimpanzees and *RGD* (which display high levels of linkage disequilibrium) than between chimpanzees and *SGD*, as a result of genetic drift.

Actually, the idea that Western chimpanzees underwent a substantial reduction in population size has been supported by analysing other parts of the genome. First, studies on both autosomal genes and whole genome sequences [44, 66, 67, 70, 86–88] have indicated that Western chimpanzees are generally less diverse than the other *Pan* sub-species, which sustains the hypothesis of several past bottlenecks in the former [44, 79]; second, Western chimpanzees’ genomic diversity has been found to fall within the average observed for Non-African human populations, which show a much lower genetic diversity than African populations (Fig. 1b of [44]). Therefore, although *MHC* genes are known to be targets of natural selection, our study reveals that traces of past bottlenecks that impacted non-*MHC* genes are detectable when analysing the genetic diversity patterns of *Patr* genes, and more particularly that of the four loci *DPB1*, *DQB1, C* and *A*.

### Signatures of natural selection on *Patr* and *HLA* loci

At the other three *MHC* loci (*DQA1*, *DRB1* and *B*), the genetic diversity observed in Western chimpanzees does not simply mirror that of human populations that likely underwent rapid genetic drift (*RGD*). Indeed, the nucleotide diversity observed in chimpanzees is either similar to or greater than (significantly at locus *B*) that found in human populations with very diverse demographic histories, e.g. in Africa and Europe (Fig. 2, central pane and Additional Figure S3). Furthermore, chimpanzees exhibit both high nucleotide diversity and low heterozygosity compared to human populations at locus *B*, while the reverse (i.e. low nucleotide diversity and high heterozygosity) is found at loci *A* and *DPB1*. The differences observed between these genes (*DQA1*, *DRB1* and *B*) and the others (*DPB1*, *DQB1*, *C* and *A)* is thus probably due to more complex mechanisms involving not only demography (as described above) but also natural selection, i.e. distinct susceptibilities of different *Patr* genes to pathogenic environments. This is not contradictory with the fact that we did not detect significant departures from selective neutrality for *Patr* genes in the studied chimpanzee cohorts, as our simulations showed that these results could be due to type II errors.

To better understand how the *MHC* polymorphism could have evolved in chimpanzees under simultaneous demographic and selective forces, we must first consider which kinds of natural selection may have targeted different *Patr* genes. According to the scenario that was initially proposed by de Groot et al. [55], a specific mechanism would have affected substantially the *MHC* genetic profile of chimpanzees, namely a strong selective sweep owing to the action of a viral pathogen (the simian form of *HIV*, i.e. *SIV* or a related retrovirus) decimating this species ~ 2 to 3 million years ago, followed by a second bottleneck in the Western subspecies [44]. As a consequence, many *Patr* class I alleles would have been lost and the only surviving individuals would have been those carrying alleles providing resistance to the involved pathogen [55, 56]. This loss of diversity would have specifically affected the *Patr-A* gene, because at this locus all alleles of a single lineage, *A2*, were virtually lost, but also the *Patr-B* and -*C* genes, based on molecular evidence at intron and *MIC* regions (see Background above). Actually, as the selective sweep that affected *Patr* genes had apparently been quite substantial, we would have expected significantly *lower* (rather than similar) levels of *MHC* genetic diversity in Western chimpanzees than in small isolated human populations that started to lose diversity much more recently (i.e. at most since modern human populations left their homeland in sub-Saharan Africa). We however have to consider here that chimpanzees had a long time to restore genetic diversity by expanding again demographically after the bottleneck(s) that affected them well before the emergence of modern humans.

Besides a selective sweep, however, balancing selection (in the form of heterozygote advantage) is another mechanism that did affect the evolution of *Patr* genes. Indeed, *MHC* genes were found to present strong signals of balancing selection in all great apes’ lineages [89]. Moreover, this kind of selection explains the sharing of ancient *MHC* lineages by humans and chimpanzees at loci *DQB1*, *DQA1*, *DRB1*, *C* and *A* [5, 16, 73, 90–92]. Actually, many works suggest that *MHC* genes are potential targets of both directional (selective sweep) and balancing selection [78, 93, 94].

### Evolution of *Patr* genes’ diversity: tentative scenarios

Taking the different evolutionary mechanisms mentioned above into account, i.e. mutational/recombination events generating molecular diversity, as well as demographic processes and distinct kinds of natural selection increasing or decreasing the levels of genetic diversity, the results uncovered by the present study support original scenarios for the evolution of *Patr* genes.

**Fig. 3:**
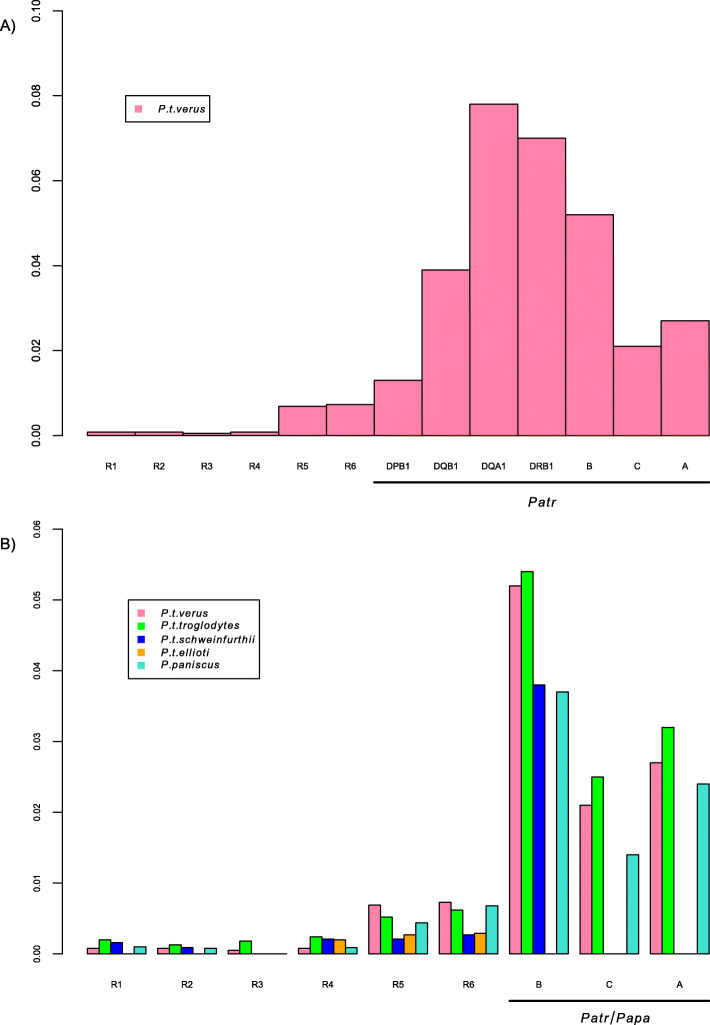
Nucleotide diversity at *MHC* loci and other genomic regions in Western chimpanzees (A) and in different sub-species of chimpanzees and bonobos (*P. paniscus*) (B). R1: Non-coding autosomal regions [66]; R2: Non-coding autosomal regions [67]; R3: Xq13.3 [95]; R4: Non-coding autosomal regions [82]; R5: Mitogenome [82]; R6: Mitogenome [54]; *Patr/Papa-B, C, A*: average nucleotide diversity for genes *Patr/Papa-B, −C, −A:* this study, [61, 62]. No data is available for R1, R2 and R3 in *P.t.ellioti*, for R3 in *P.paniscus* and for R3 in *P.t schweinfurthii*. Values are given in Additional Table S10

For class I genes, we principally hypothesize that the genetic diversity of *Patr-A* and *Patr-B* regenerated when Western chimpanzees expanded demographically (although to a small extent) after the bottlenecks that occurred, first, ~ 2 to 3 million years ago in the ancestors of chimpanzees and bonobos [55, 56] and, later on, about 500,000 years ago in the likely differentiated Western chimpanzee subspecies [44]. This idea finds good support in the equivalent amounts of *Patr* class I nucleotide diversity found in Western (*P.t.verus*) and Central (*P.t.troglodytes*) chimpanzees (Fig. 3), in spite of the latter having experienced the least severe population bottleneck among all *Pan* subspecies [44].

This recovery of genetic variation would have occurred, however, through distinct mechanisms and with distinct intensities at the two loci *Patr-A* and *Patr-B*. At *Patr-B*, recombination and/or gene conversion would have rapidly created new alleles and highly divergent sequences, explaining why chimpanzees, like humans, display higher nucleotide diversity at this locus than at the other class I genes. Asymmetric balancing selection, whereby heterozygotes with more divergent alleles would have an advantage [21, 96] would have also acted on *Patr-B*, as this type of selection also tends to increase nucleotide diversity [97]. Noteworthy is the fact that particular *HLA-B* alleles have been positively selected in African human populations in response to *Plasmodium falciparum* malaria [26] and that functionally similar alleles have recently been identified in bonobos which live in an area with a high prevalence of this parasite [27]. If we assume that common chimpanzees underwent similar responses to pathogens, locus *Patr-B* (like *HLA-B* in humans [26]) would have been affected by a (relatively) soft selective sweep whereby several alleles have been positively selected, thus explaining both the high cumulated frequency of three *Patr-B* alleles (see Supplementary Text) and the high values of heterozygosity and allelic richness found at this locus. By contrast, at *Patr-A* new variants would have primarily been generated by point mutations that accumulate at slow rates during evolution, which may explain the low nucleotide diversity observed at this locus. Nevertheless, the high heterozygosity found at *Patr-A* (actually slightly higher than at *Patr-B* and *HLA-A*) suggests that heterozygous advantage also had a substantial effect on this gene after its drastic loss of diversity, possibly as an efficient way to rapidly restore a minimal immune protection despite a slow regeneration of diversity through point mutations. Interestingly, *Patr-A* molecules display a lower peptide binding repertoire than *Patr-B* and *HLA-A* [98], suggesting that they have also evolved a peptide binding site that is more promiscuous [64, 99] as a compensation for their severe loss of diversity or that promiscuous alleles were selected preferentially [31]. Finally, the genetic diversity of *Patr-A* and *Patr-B* might have evolved in concert according to a model of joint asymmetric selection as proposed for *HLA-A* and *HLA-B* [64, 100], allowing distinct levels of polymorphism to be maintained at the two loci as long as both of them have jointly ensured a sufficient immune protection.

Compared to *Patr-A* and *Patr-B*, *Patr-C* displays a lower level of nucleotide diversity in chimpanzees, like in humans. Knowing that both *Patr-C* and *HLA-C* molecules are ligands for killer-cell immunoglobulin-like receptors (KIR) [101, 102], the interaction of HLA and KIR molecules being crucial to regulate the killer function of natural killer cells [103], *Patr-C* molecules were probably submitted to similar functional constraints as *HLA-C*, resulting in substantial directional and/or purifying selection. However, contrary to *Patr-DQB1*, for which we suppose the same kinds of selection as for *Patr-C* (see below), the strong linkage disequilibrium that characterizes the *B ~ C* loci pair in chimpanzees and humans might have attenuated the opposite effects of balancing and positive/purifying selection impacting loci *B* and *C*, respectively.

For class II genes, our results also indicate distinct evolutionary histories for the different loci. As *MHC* class II genes more specifically respond to parasitic and bacterial infections, they would have been less directly impacted by the viral epidemic proposed in [55, 56]. Moreover, the selection criteria are also less strict as class II genes are generally more promiscuous binders and select longer peptides for binding. Nevertheless, like *Patr-A*, it is likely that *Patr-DRB1* underwent a substantial selective sweep reducing the number of allele lineages, as inferred from its much lower allelic richness compared to *Patr-B* and in agreement with the apparent loss of all alleles belonging to the DRB1*04 lineage [5]. Such selection would have been mostly independent from that affecting class I genes – i.e. possibly involving other pathogens – since global linkage disequilibrium is not significant between *DRB1* and class I genes. Also, because *Patr-DRB1* evolves through recombination and/or gene conversion, its putative loss of diversity in the past would have been followed, as proposed above for *Patr-B,* by a rapid regeneration of nucleotide diversity, which is particularly high at this locus (Fig. 2). Note also that in chimpanzees, *MHC* class II diversity is particularly high at the haplotype level thanks to inter-locus recombinations despite important loss of variation at single genes due to the past selective sweep [104].

*Patr-DQA1* and *Patr-DQB1* exhibit contrasting levels of nucleotide diversity and heterozygosity, high for *DQA1* and low for *DQB1* (note, however, that *Patr-DQA1* data were only available for one cohort of chimpanzees, BPRC^wb^), despite the fact that these two genes are in strong linkage disequilibrium and encode the two complementary chains of the Patr-DQ molecules. Among all loci tested, *Patr-DQB1* is actually the most divergent to its orthologue in humans for these two indexes (Fig. 2). Studies have stressed the fact that DQ molecules evolve under purifying selection due to strong functional constraints and with a limited dynamic of evolution in both humans and chimpanzees [56, 105]. The low diversity found at *Patr-DQB1*, with a single allele (DQB1*03:02) reaching a frequency above 60% in the BPRC^wb^ cohort (Additional Figure S4), would indicate a stronger constraint on the β chain. By contrast, *Patr-DQA1* would have evolved by maintaining several alleles (although a limited number, like at *DQB1*) at more even frequencies, as also observed for *HLA-DQA1* in human populations [21, 106]. Based on our results, we also hypothesize that the very high nucleotide diversity observed at *Patr-DQA1* (the highest of all studied loci) results from a molecular evolution mainly characterized by recombination and/or gene conversion rather than point mutations.

Finally, the low nucleotide diversity (and, to a lesser extent, allelic richness) found at *Patr-DPB1* is comparable to that observed at *HLA-DPB1* in small-sized and isolated populations that likely experienced rapid genetic drift (such as Australian Aborigines and Amerindians), although this is not the case when looking at heterozygosity (Additional Figure S3). These results suggest an effect of balancing selection in the form of heterozygous advantage (explaining the high level of heterozygosity) combined with a slow generation of diversity through point mutations (explaining the low nucleotide diversity falling at the opposite of what is observed for *Patr-DQA1*), as suggested for *Gogo-DPB1* in gorillas [107]. Interestingly, the low nucleotide diversity observed at *DPB1* appears to be rather close to that observed at neutral genomic regions, although the whole *Patr* region is clearly exceptionally diverse in this respect (Fig. 3 and Additional Table S10).

**Fig. 4:**
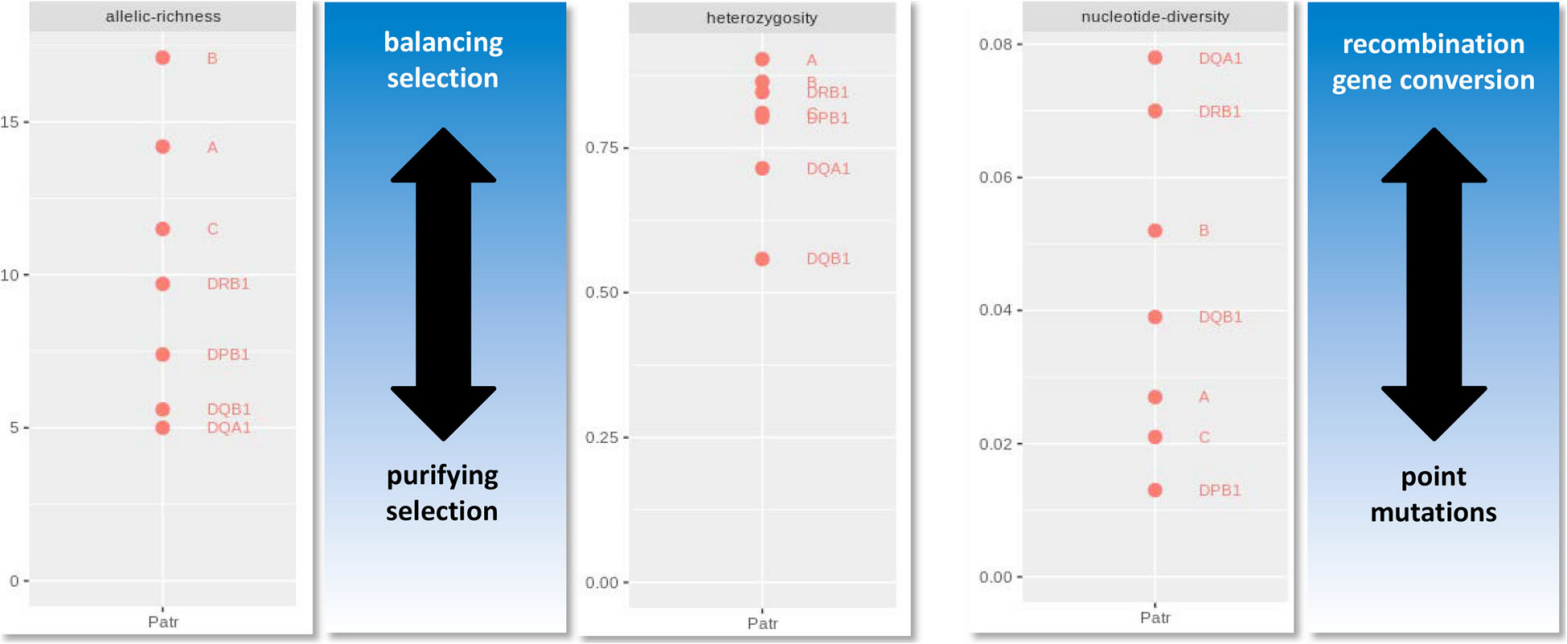
Schematic representation of the evolutionary mechanisms explaining the genetic diversity observed in *Patr* genes. For each diversity index, the *Patr* loci are plotted according to the values given in Table 3 for the pooled cohort of chimpanzees. The pooled cohort includes all cohorts except Texas^cb^

The main mechanisms that would explain the evolution of the different *Patr* genes after the ancient bottlenecks that affected Western chimpanzees are illustrated in Fig. 4.

## Conclusions

By revealing similar patterns of genetic diversity and linkage disequilibrium in Western chimpanzees and humans across the main *MHC* loci, our study suggests that these genes have been shaped by analogous mechanisms in both species despite several million years of independent evolution. This led us to conclude that the *MHC* region and the evolutionary mechanisms shaping it have been highly conserved in the human and chimpanzee lineages. Our work also uncovered deep similarities between Western chimpanzees and smaller, isolated human populations most likely having undergone rapid genetic drift, independently of their geographic locations and genetic backgrounds, supporting a substantial effect of limited population sizes on *MHC* evolution in both species. We then proposed plausible scenarios for the molecular evolution of each *Patr* gene taking into account the strong selective sweep(s) that affected *Patr* genes after the ancient bottlenecks of Western chimpanzees that, curiously enough, did not substantially deplete their levels of *MHC* genetic diversity. These scenarios suggest that several *Patr* genes recovered allelic and/or nucleotide diversity after these bottlenecks thanks to the action of both balancing selection (*DRB1, B, A*) and rapid generation of polymorphism through recombination and/or gene conversion (*DRB1, B*). On the other hand, other loci kept a rather low diversity due to stronger directional or purifying selection and/or a slower process of molecular diversification through point mutations (*DQB1, C*), and some mixed processes also likely occurred (*DPB1*, *DQA1*). The possibility to substantially regenerate a high genetic diversity after a bottleneck, as originally proposed for *Patr* genes in this study, is essential for genes involved in immunity, like those of the *MHC* complex. Indeed, such a process is likely to restore the potential of a population to resist multiple infectious diseases and may thus be decisive for the long-term survival of critically endangered species like the chimpanzee.

## Methods

### Chimpanzee cohorts

The chimpanzee data include both wild-born (wb) and captive-born (cb) Western chimpanzees (*P.t.verus* called *chimpanzees* hereafter) for which *Patr* analyses were previously published. The available data include four cohorts:

1. BPRC^wb^, consisting of 29 wild-born individuals captured in Sierra-Leone in the late seventies, who further founded the colony that was originally housed at the Biomedical Primate Research Centre (BPRC) [16, 58, 99]. According to mitochondrial and segregation analyses [108], all individuals appear to be unrelated.
2. Yerkes^cb^, consisting of 22 captive-born individuals from US institutions [59, 109, 110]. Relatedness between animals is unknown.
3. Texas^cb^, consisting of 23 captive-born individuals housed in US institutions [60, 111]. However, contrary to Yerkes^cb^, this cohort may contain animals from different sub-species and/or hybrid animals (personal communication from the authors of [60]).
4. Kuma^wb^, consisting of 19 wild-born individuals (of unknown origin, captured in the seventies) who were previously housed in research institutions in Japan and were further retired in the Kumamoto Primate Park, Japan [112–114]. Relatedness between animals is unknown.

We arranged the samples in two ways for the analyses: a) by considering separately the four cohorts defined above; and b) by grouping the individuals from BPRC^wb^, Yerkes^cb^ and Kuma^wb^ within a single cohort (called the “pooled cohort” hereafter). We did not include Texas^cb^ in the pooled cohort because of uncertainties regarding the represented sub-species.

The detailed information of each chimpanzee cohort is given in Additional Table S11.

### Human populations

The human data are a subset of 50 to 89 population samples (depending on the locus) taken from the *HLA*-typed populations analysed in [21]. They represent 10 geographical regions (North Africa, South Africa, North America, South America, Europe, South-East Asia, North-East Asia, South-West Asia, Australia and Pacific). Based both on a previous paper using most of the same population samples as in this study [100] and on additional ethnological information [50], we defined each population as either RGD (meaning *rapid genetic drift*) or SGD (meaning *slow genetic drift*). RGD include small and isolated populations from different continents, mostly Indigenous populations from North and South America, Taiwan, Indonesia, Melanesia and Australia as well as populations from the Saharan region (e.g. Berber speaking) and hunter-gatherers from Central Africa, all other population being classified as SGD. All human populations were analysed separately from each other (i.e. never pooled as a single dataset but considered as multiple populations taken together when reporting the results) in the whole study. The detailed information of each human population sample is given in Additional Table S12.

### MHC data

For both chimpanzee cohorts and human populations, the *MHC* data consist of multi-locus genotypes (including loci *A*, *B*, *C*, *DRB1*, *DQA1*, *DQB1* and/or *DPB1*) composed of alleles defined at the 2nd field level of resolution according to the official nomenclatures of the IPD-MHC [115, 116] and IPD-IMGT/HLA [17] databases for *Patr* and *HLA*, respectively. At this resolution level, the alleles differ by one or more nucleotide substitutions that change the amino acid sequence of the *MHC* protein. Moreover, because these data result from exons 2 and 3 (for class I) or exon 2 (for class II) molecular typings, the assessed variation was restricted to the PBR (class I: full exons 2 and 3 sequences; class II: full exon 2 sequences).

A summary of the data used in this study is presented in Table 6.

**Table 6:** Summary of the chimpanzee and human population data at* Patr* and *HLA* genes, respectively

Chimpanzees (individual and pooled cohorts)		*DPB1*	*DQB1*	*DQA1*	*DRB1*	*B*	*C*	*A*
*N*	BPRC^wb^	25	29	29	29	29	29	28
	Yerkes^cb^					22	22	22
	Texas^cb^		16		17	23		23
	Kuma^wb^	19	19		17			
	Pooled cohort^a^	44	48	29	46	51	51	50
Humans (multiple populations)		*DPB1*	*DQB1*	*DQA1*	*DRB1*	*B*	*C*	*A*
*k*	Africa	7	19	8	12	11	8	11
	Europe	14	21	15	16	6	3	6
	Asia	9	12	6	33	38	30	36
	N/S America	11	19	17	18	9	5	9
	Australia	3	2	2	3	4	4	4
	Pacific	6	6	4	6	5	4	8
	Other^b^	0	0	0	1	7	5	7
$\overline{N}$*(s.d)*		87.8 (45.2)	100.6 (55.3)	90.7 (45.5)	105.5 (111.8)	124 (134.6)	127.9 (141.1)	127.6 (133.6)

N: sample size (in number of individuals); k: number of populations per geographic region; $\overline{N}$: mean sample size (in number of individuals); s.d.: standard deviation. The order of loci corresponds to their position on the chromosome from centromere (left) to telomere (right)

^a^does not include Texas^cb^ (see Text)

^b^Populations were allocated to one of six geographic regions, and we considered an additional category Other for known admixed populations

### Statistical analyses

#### Allele frequencies and Hardy-Weinberg equilibrium

We estimated allele frequencies with an EM algorithm, the Gene-Counting Expectation Maximisation algorithm implemented in [117]. These estimates can be considered as population frequencies if Hardy-Weinberg equilibrium (HWE) is satisfied. We thus tested HWE in all populations by using a Likelihood Ratio Test (LRT) that compares the likelihood of frequencies estimated under HWE to the likelihood of those estimated under an inbreeding model.

#### Genetic diversity

We determined genetic diversity within each chimpanzee cohort or human population by using three different statistics: allelic richness *ar*, expected heterozygosity *h*, and nucleotide diversity *π*:

1. Allelic richness *ar* was estimated by the number of alleles expected in a population sample of size equal to the rarefaction size *2n* (i.e. the size of the smallest sample of *n* individuals at this locus) [118] as:
  $$ ar=\sum \limits_{\mathrm{i}=1}^{\mathrm{k}}1-\frac{\left(\genfrac{}{}{0pt}{}{2\mathrm{N}-{\mathrm{N}}_{\mathrm{i}}}{2\mathrm{n}}\right)}{\left(\genfrac{}{}{0pt}{}{2\mathrm{N}}{2\mathrm{n}}\right)} $$

where *k* is the number of alleles in the sample, *2n* the rarefaction size and *N*_*i*_ the number of occurrences of the *i*^th^ allele among the *2 N* sampled genes. Using this index is particularly appropriate when highly polymorphic genes like *MHC* are studied in samples of small sizes. Rarefaction sizes (2*n*) were 50 for *A*, 58 for *B*, 56 for *C*, 60 for *DPB1*, 58 for *DQA1*, 66 for *DQB1* and 52 for *DRB1* when allelic richness was estimated on the pooled cohort of chimpanzees and the different human population samples, and 44 for *A*, 44 for *B*, 44 for *C*, 38 for *DPB1*, 58 for *DQA1*, 32 for *DQB1* and 34 for *DRB1* when the four cohorts of chimpanzees were considered separately.

2. Expected heterozygosity *h* (equivalent to Nei’s gene diversity, [119]) within a sampled population at HWE was computed according to:
  $$ h=1-\sum \limits_{\mathrm{i}=1}^{\mathrm{k}}{{\mathrm{p}}_{\mathrm{i}}}^2 $$

where *k* is the number of alleles and *p*_*i*_ the frequency of the *i*th allele in the sample. Expected heterozygosity is not necessarily correlated to allelic richness since the latter is only influenced by the number of alleles and not by their frequency; for example, identical allelic richness may be observed in populations showing dissimilar heterozygosity (i.e. high heterozygosity due to the presence of many intermediate frequency alleles, as expected under balancing selection, or low heterozygosity due to the presence of one very frequent and many rare alleles, as expected under purifying selection).

3. Contrary to the expected heterozygosity, nucleotide diversity π takes into account the number of nucleotide differences between alleles [119]. To compute this index, a DNA sequence (class I: exon 2 and 3; class II: exon 2) was first assigned to each allele by using the IPD/MHC and IPD/IMGT-HLA resources [115, 116, 120]. Nucleotide diversity was then estimated as:
  $$ \pi =\frac{\sum_{i=1}^k\sum \limits_{j<i}{p}_i{p}_j{d}_{ij}}{L} $$

where *k* is the number of alleles, *L* the number of sites in the sequence, *p*_*i*_ and *p*_*j*_ the frequencies of the *i*^th^ and *j*^th^ allele in the sample, respectively, and *d*_*ij*_ the number of nucleotide differences observed between alleles *i* and *j*. Nucleotide diversity is not necessarily correlated to expected heterozygosity; for example, identical heterozygosity may be observed in populations showing distinct genetic profiles where alleles are either molecularly very close (i.e. due to their slow diversification through rare point mutations) or molecularly very distant (i.e. due to their rapid diversification through recombination and/or gene conversion).

The three indices described above complement each other as they convey a different information on the genetic diversity observed within a given cohort or population.

#### Selective neutrality

To assess whether MHC genes are significantly submitted to selective pressures or behave as neutral markers, we searched for signals of natural selection by applying the Slatkin’s version of the Ewens-Watterson selective neutrality test (named EWS test thereafter) based on allele frequencies [121–124] as implemented in [117]. The *p*-values obtained through the resampling process were adjusted for multiple testing using the False Discovery Rate (FDR) method [125]. The tests were done without prior assumptions, thus two-tailed rejection at the 5% level either occurs above 97.5% for excess of homozygotes or below 2.5% for excess of heterozygotes.

#### Linkage disequilibrium

As our study explores the genetic diversity at multiple *MHC* loci, we estimated both global linkage disequilibrium and proportions of haplotypes in significant linkage disequilibrium for all pairs of loci for which data were available. The assessment of global linkage disequilibrium was performed by means of a resampling procedure (named PRS, for Parametric Resampling Schema, hereafter) generating an empirical distribution for a likelihood ratio test (LRT) statistic based on the likelihood of allele and haplotype frequency estimates, the final result being the percentile of the observed LRT statistic (PRS) in the empirical distribution [106, 126] . Haplotypes in significant linkage disequilibrium were determined by a χ2 test (see Supplementary Text).

#### Genetic distances

We compared the *Patr* frequency distributions between each pair of chimpanzee cohorts by computing Prevosti’s genetic distances [127] according to:

$$ {D}_{P,Q}=\frac{1}{2}\sum \limits_{i=1}^k\left|{p}_i-{q}_i\right| $$

where *p*_*i*_ and *q*_*i*_ represent the frequencies of allele *i* in populations *P* and *Q*, respectively. The proportion of shared frequencies between cohorts was then estimated as the complement to 1 of Prevosti’s distance given in percentages.

All frequency estimations and statistical analyses based on allele frequencies were performed using the *hla-net* (*www.hla-net.eu*) Gene [rate] tools [117]. *Arlequin* 3.5 [128] and *Fstat* [129] were used to estimate nucleotide diversity and allelic richness, respectively. When necessary, *p*-values were adjusted using Holm’s correction [130].

#### Computer simulations

We checked the robustness of our results by controlling for the great discrepancy in sample sizes between chimpanzee cohorts and human populations through computer simulations using a resampling procedure. For each human population sample and each locus, we randomly drew 1000 sub-samples of the same size as the pooled cohort of chimpanzees (i.e. *N* = 44 for *DPB1*, 48 for *DQB1*, 29 for *DQA1*, 46 for *DRB1*, 51 for *B*, 51 for *C* and 50 for *A*) on which we tested Hardy-Weinberg equilibrium, we estimated the 3 diversity indices, we applied the selective neutrality test and we assessed linkage disequilibrium.

## Supplementary information

**Additional file 1: Additional Table S1.** Allele frequencies and results of HWE and SEW tests at each Patr locus in the pooled and the four cohorts of chimpanzees.

**Additional file 2: Additional Table S2.** List of human population samples per HLA locus. For each sample, its population name, it country and region, the population size as well as heterozygosity (H) and nucleotide diversity (π), allelic richness (ar) and the results of the Slatkin Ewens-Watterson test of neutrality and Hardy-Weinberg (HW) equilibrium are given. The table presents also the results of the simulations for diversity indices (average value and standard deviation, and the confidence interval at 95% on the 1000 sub-samples); the results of the simulations for Hardy-Weinberg test (proportion of rejections of HW test in the 1000 sub-samples); the results of the simulations for Ewens-Watterson test (proportion of rejections of Ewens-Watterson test (rejections for both an excess of homozygotes or of heterozygotes) in the 1000 sub-samples).

**Additional file 3: Additional Table S3.** Genetic diversity at different *Patr* genes in chimpanzees (multiple cohorts and in the pooled cohort). *ar: allelic richness; H: heterozygosity; П: nucleotide diversity; −: data not available; the values of this table were used in* Fig. 2*.*

**Additional file 4: Additional Table S4**. Test of difference between diversity indexes in chimpanzee cohorts and in human populations subdivided into those that likely followed rapid /or slow genetic drift (RGD and SGD). For each locus, the *p*-value of the Wilcoxon test is given. p-value in bold are significant at 5% level, and * indicates significant results after correction for the number of loci.

**Additional file 5: Additional Table S5.** Results of Global Linkage Disequilibrium (GLD) PRS significance test^1^ between different pairs of MHC loci in individual chimpanzee cohorts and in the pooled cohort.

**Additional file 6: Additional Table S6.** Proportion of haplotypes in significant linkage disequilibrium (LD) in chimpanzees (BPRC cohort) and humans (multiple populations, further subdivided into RGD and SGD populations).

**Additional file 7: Additional Table S7.** Linkage disequilibrium between pairs of alleles in chimpanzees.

**Additional file 8: Additional Table S8.** Global linkage disequilibrium estimated by PRS test between pairs of loci and proportion of haplotypes in significant linkage disequilibrium for each human population sample.

**Additional file 9: Additional Table S9.** Results of the simulations on linkage desequilibrium. For each population, the number of simulated samples presenting global linkage disequilibrium (according to the LRT and PRS tests) as well as the average, the standard deviation and the 95% confidence interval of the proportion of haplotypes in linkage disequilibrium in the simulated samples. The values for the original population sample are given as a reminder.

**Additional file 10: Additional Table S10.** Nucleotide diversity (П) at *Patr* loci and other genomic regions in chimpanzees (*Pan troglodytes* subspecies) and bonobos (*Pan paniscus*).

**Additional file 11: Additional Table S11**. List of individuals of the four cohorts of chimpanzees. For each chimpanzee, its name, birth status (wild-born or captive-born), its origin (as defined in the publication), its country of origin (if known), its gender, its sub-species, the publication and the Patr gene studied is given.

**Additional file 12: Additional Table S12.** List of human population samples per HLA locus. For each sample, population name, country and region of origin and whether the population has likely been submitted to rapid genetic drift are given. Sample size per locus is also given.

**Additional file 13: Additional Table S13.** Pairs of genotypes per individual and locus.

**Additional file 14: Additional Table S14.** Number of individuals for each genotype.

**Additional file 15. Additional Table S15.** Percentages of shared allelic frequencies between chimpanzee cohorts at different *Patr* loci.

**Additional file 16: Additional Table S15**. Percentages of shared allelic frequencies between chimpanzee cohorts at different *Patr* locus.

**Additional file 17: Additional Figure S1.** Genetic diversity in chimpanzees (pooled cohort) and humans (multiple populations). A) Allelic richness B) heterozygosity, C) nucleotide diversity at each locus under study for the pooled cohort of chimpanzees (in red empty circle) and for the human populations (in blue) represented as violin plots; An average number of k = 70 (s.d 15.9) human population samples of average size *N* = 109.2 (s.d 17.31) were used. The width of the violin varies so as to represent the probability density of the data, the thick blue bar in the centre represents the interquartile range, the thin black line extended from it represents the 95% confidence intervals, and the green dot is the median. The MHC loci are presented according to their position on the chromosome from the centromere (left) to the telomere (right).

**Additional file 18: Additional Figure S2.** Genetic diversity in chimpanzees (pooled cohort) and humans (multiple populations and simulated populations). A) Allelic richness B) heterozygosity, C) nucleotide diversity at each locus under study for the pooled cohort of chimpanzees (in black filled circle) and for the human populations represented as violin plots. Diversity values of each human population sample are plotted as a violin plot in red; Simulated diversity values (1000 samples of the same size per locus as the pooled cohort of chimpanzees) are plotted as a violin plot in blue. The width of the violin varies so as to represent the probability density of the data, the thick blue bar in the centre represents the interquartile range, the thin black line extended from it represents the 95% confidence intervals, and the green dot is the median. The MHC loci are presented according to their position on the chromosome from the centromere (left) to the telomere (right).

**Additional file 19: Additional Figure S3.** Genetic diversity in chimpanzees (multiple cohorts) and humans (multiple populations) averaged by geographic regions. A) Allelic richness B) heterozygosity, C) nucleotide diversity at each locus under study for each cohort of chimpanzees and for the human populations where samples are grouped by geographic regions; The values calculated for each chimpanzee cohort are indicated by filled and unfilled shapes in red for cohorts of wild-born and captive-born chimpanzees, respectively; for the human populations, each region is represented by a triangle of different colour as defined in legend. The MHC loci are presented according to their position on the chromosome from the centromere (left) to the telomere (right).

**Additional file 20: Additional Figure S4.** Allele frequency distributions for class I and class II loci in the cohorts of chimpanzees including the pooled cohort. 1: locus B, 2: locus C, 3: locus A, 4: locus DPB1, 5: locus DQB1, 6: locus DQA1, and 7: locus DRB1. Alleles are represented by different colours as defined in the legend. Colours in the legend follow the same order as allele frequencies in the plot. Values are in Additional Table S3.

## Data Availability

The online version of this article contains supplementary material, which is available to authorized users. Individual chimpanzee details (cohort, number, name, birth status, origin, country of origin, gender, species and publication) alongside MHC genotypes for the chimpanzee cohorts are available in additional files Additional Table S11 and Additional Table S13. Description and results on human populations are in Additional Table S12 and Additional Table S2.

## Abbreviations

MHC: Major Histocompatibility Complex
HLA: Human Leukocyte Antigen
Patr: Name of the MHC region in chimpanzees, for ***Pa****n* ***tr****oglodytes*
Papa: Name of the MHC region in bonobos, for ***Pa****n* ***pa****niscus*
Mamu: Name of the MHC region in rhesus monkeys, for ***Ma****caca* ***mu****latta*
Popy: Name of the MHC region in Bornean orangutans, for ***Po****ngo* ***py****gmaeus*
Gogo: Name of the MHC region in gorillas, for ***Go****rilla* ***go****rilla*
Myr: Million years
DAA: Divergent Allele Advantage
P. t.: *Pan troglodytes*
P. p.: *Pan paniscus*
wb: Wild born
cb: Captive born
BPRC: Biomedical Primate Research Centre
RGD: Rapid Genetic Drift
SGD: Slow Genetic Drift
PBR: Peptide Binding Region
EM: Expectation Maximization
HWE: Hardy-Weinberg Equilibrium
LRT: Likelihood Ratio Test
ar: Allelic richness
h: Heterozygosity
π: Nucleotide diversity
EWS: Ewens-Watterson-Slatkin
FDR: False Discovery Rate
PRS: Parametric Resampling Schema
GLD: Global Linkage Disequilibrium
LD: Linkage Disequilibrium
KIR: Killer-cell Immunoglobulin-like Receptors
Kb: Kilobases
s.d: Standard deviation
