# Supplementary material for: Similar patterns of genetic diversity and linkage disequilibrium in Western chimpanzees (Pan troglodytes verus) and humans indicate highly conserved mechanisms of MHC molecular evolution
Source: BMC Evol Biol. 2020 Sep 15;20:119. doi: 10.1186/s12862-020-01669-6 (PMC7491122; doi:10.1186/s12862-020-01669-6)
Supplement: Supplementary file 3 — Additional file 3: Additional Table S3. Genetic diversity at different Patr genes in chimpanzees (multiple cohorts and in the pooled cohort). ar: allelic richness; H: heterozygosity; П: nucleotide diversity; −: data not available; the values of this table were used in Fig. 2. [file 12862_2020_1669_MOESM3_ESM.docx]

**Additional Table S3:** **Genetic diversity at different *Patr* genes in chimpanzees (multiple cohorts and in the pooled cohort).**

*ar: allelic richness; H: heterozygosity; П: nucleotide diversity; -: data not available; the values of this table were used in Figure 2.*

|  | *ar* | | | | |  | *H* | | | | |  | *П* | | | | |
| --- | --- | --- | --- | --- | --- | --- | --- | --- | --- | --- | --- | --- | --- | --- | --- | --- | --- |
| Locus | BPRC^wb^ | Yerkes^cb^ | Texas^cb^ | Kuma^wb^ | Pooled cohort |  | BPRC^wb^ | Yerkes^cb^ | Texas^cb^ | Kuma^wb^ | Pooled cohort |  | BPRC^wb^ | Yerkes^cb^ | Texas^cb^ | Kuma^wb^ | Pooled cohort |
| *DPB1* | 7.51 | *-* | *-* | 5 | 7.4 |  | 0.811 | *-* | *-* | 0.749 | 0.803 |  | 0.013 | *-* | *-* | 0.012 | 0.013 |
| *DQB1* | 4.41 | *-* | 6 | 4.68 | 5.6 |  | 0.509 | *-* | 0.783 | 0.619 | 0.558 |  | 0.036 | *-* | 0.042 | 0.042 | 0.039 |
| *DQA1* | 5 | *-* | *-* | *-* | 5 |  | 0.715 | *-* | *-* | *-* | 0.715 |  | 0.078 | *-* | *-* | *-* | 0.078 |
| *DRB1* | 9.19 | *-* | 13 | 8 | 9.7 |  | 0.829 | *-* | 0.891 | 0.822 | 0.846 |  | 0.075 | *-* | 0.094 | 0.075 | 0.07 |
| *B* | 10.15 | 17 | 14.65 | *-* | 17.1 |  | 0.801 | 0.879 | 0.842 | *-* | 0.864 |  | 0.048 | 0.055 | 0.046 | *-* | 0.052 |
| *C* | 7.46 | 12 | *-* | *-* | 11.5 |  | 0.766 | 0.826 | - | *-* | 0.810 |  | 0.022 | 0.019 | *-* | *-* | 0.021 |
| *A* | 12.3 | 14 | 12.74 | *-* | 14.2 |  | 0.876 | 0.899 | 0.882 | *-* | 0.903 |  | 0.024 | 0.03 | 0.027 | *-* | 0.027 |
