# Supplementary material for: Similar patterns of genetic diversity and linkage disequilibrium in Western chimpanzees (Pan troglodytes verus) and humans indicate highly conserved mechanisms of MHC molecular evolution
Source: BMC Evol Biol. 2020 Sep 15;20:119. doi: 10.1186/s12862-020-01669-6 (PMC7491122; doi:10.1186/s12862-020-01669-6)
Supplement: Supplementary file 4 — Additional file 4: Additional Table S4. Test of difference between diversity indexes in chimpanzee cohorts and in human populations subdivided into those that likely followed rapid /or slow genetic drift (RGD and SGD). For each locus, the p-value of the Wilcoxon test is given. p-value in bold are significant at 5% level, and * indicates significant results after correction for the number of loci. [file 12862_2020_1669_MOESM4_ESM.docx]

**Additional Table S4**: Test of difference between diversity indexes in chimpanzee cohorts and in human populations subdivided into those that likely followed rapid /or slow genetic drift (RGD and SGD). For each locus, the p-value of the Wilcoxon test is given. p-value in bold are significant at 5% level, and * indicates significant results after correction for the number of loci.

|  | p-values | | | | | |
| --- | --- | --- | --- | --- | --- | --- |
|  | Allelic richness | | Heterozygosity | | Nucleotide diversity | |
|  | RGD | SGD | RGD | SGD | RGD | SGD |
| DPB1 | 0.732 | **0.0049*** | 0.1052 | 0.485 | 0.4706 | **0.0209** |
| DQB1 | 0.4576 | **0.0001*** | 0.4469 | **0.0051*** | 0.7816 | **0.004*** |
| DRB1 | 0.8955 | **0.0009*** | 0.6751 | **0.0202** | **0.032** | 0.733 |
| B | 0.4618 | **0.0008*** | 1 | **0.0046*** | **0.0074*** | **0.0349** |
| C | 0.48 | **0.0171** | 1 | **0.0202** | **0.0448** | **0.0185** |
| A | **0.0194** | 0.3865 | **0.0159** | 0.865 | 0.53 | **0.00388*** |
