# Supplementary material for: Similar patterns of genetic diversity and linkage disequilibrium in Western chimpanzees (Pan troglodytes verus) and humans indicate highly conserved mechanisms of MHC molecular evolution
Source: BMC Evol Biol. 2020 Sep 15;20:119. doi: 10.1186/s12862-020-01669-6 (PMC7491122; doi:10.1186/s12862-020-01669-6)
Supplement: Supplementary file 5 — Additional file 5: Additional Table S5. Results of Global Linkage Disequilibrium (GLD) PRS significance test1 between different pairs of MHC loci in individual chimpanzee cohorts and in the pooled cohort. [file 12862_2020_1669_MOESM5_ESM.docx]

Additional Table S5: Results of Global Linkage Disequilibrium (GLD) PRS significance test^1^ between different pairs of MHC loci in individual chimpanzee cohorts and in the pooled cohort.

|  |  | Chimpanzees^2^ | | | | | | | | | | | | | |
| --- | --- | --- | --- | --- | --- | --- | --- | --- | --- | --- | --- | --- | --- | --- | --- |
|  |  | BPRC^WB^ | |  | Texas^CB^ | |  | Yerkes^CB^ | |  | Kumamoto^WB^ | |  | Pooled cohort | |
|  |  | *N* | *p* |  | *N* | *p* |  | *N* | *p* |  | *N* | *p* |  | *N* | *p* |
| *DPB1~DQB1* |  | 25 | 0.106 |  |  |  |  |  |  |  | 19 | 0.774 |  | 44 | 0.755 |
| *DPB1~DQA1* |  | 25 | 0.0697 |  |  |  |  |  |  |  |  |  |  |  |  |
| *DPB1~DRB1* |  | 25 | 0.328 |  |  |  |  |  |  |  | 17 | 0.833 |  | 42 | 0.381 |
| *DPB1~B* |  | 25 | 1 |  |  |  |  |  |  |  |  |  |  |  |  |
| *DPB1~C* |  | 25 | 0.999 |  |  |  |  |  |  |  |  |  |  |  |  |
| *DPB1~A* |  | 24 | 0.982 |  |  |  |  |  |  |  |  |  |  |  |  |
| *DQB1~DQA1* |  | 29 | **< 10^-4^** |  |  |  |  |  |  |  |  |  |  |  |  |
| *DQB1~DRB1* |  | 29 | **< 10^-4^** |  | 16 | 0.726 |  |  |  |  | 17 | 0.162 |  | 46 | **< 10^-4^** |
| *DQB1~B* |  | 29 | 1 |  | 16 | 0.932 |  |  |  |  |  |  |  |  |  |
| *DQB1~C* |  | 29 | 0.975 |  |  |  |  |  |  |  |  |  |  |  |  |
| *DQB1~A* |  | 28 | 0.98 |  | 16 | 0.933 |  |  |  |  |  |  |  |  |  |
| *DQA1~DRB1* |  | 29 | **< 10^-4^** |  |  |  |  |  |  |  |  |  |  |  |  |
| *DQA1~B* |  | 29 | 1 |  |  |  |  |  |  |  |  |  |  |  |  |
| *DQA1~C* |  | 29 | 1 |  |  |  |  |  |  |  |  |  |  |  |  |
| *DQA1~A* |  | 28 | 0.831 |  |  |  |  |  |  |  |  |  |  |  |  |
| *DRB1~B* |  | 29 | 1 |  | 17 | 1 |  |  |  |  |  |  |  |  |  |
| *DRB1~C* |  | 29 | 0.998 |  |  |  |  |  |  |  |  |  |  |  |  |
| *DRB1~A* |  | 28 | 0.991 |  | 17 | 1 |  |  |  |  |  |  |  |  |  |
| *B~C* |  | 29 | **0.0001** |  |  |  |  | 22 | 1 |  |  |  |  | 51 | **< 10^-4^** |
| *B~A* |  | 28 | 0.931 |  | 23 | 1 |  | 22 | 1 |  |  |  |  | 50 | 1 |
| *C~A* |  | 28 | 0.892 |  |  |  |  | 22 | 1 |  |  |  |  | 50 | 1 |

^1^The resampling procedure was done with 10’000 simulations for the chimpanzee cohorts (significant results are in bold). *N*: number of individuals; *p*: p-value.

^2^The values for BPRC are from Table 5.
