# Supplementary material for: Similar patterns of genetic diversity and linkage disequilibrium in Western chimpanzees (Pan troglodytes verus) and humans indicate highly conserved mechanisms of MHC molecular evolution
Source: BMC Evol Biol. 2020 Sep 15;20:119. doi: 10.1186/s12862-020-01669-6 (PMC7491122; doi:10.1186/s12862-020-01669-6)
Supplement: Supplementary file 6 — Additional file 6: Additional Table S6. Proportion of haplotypes in significant linkage disequilibrium (LD) in chimpanzees (BPRC cohort) and humans (multiple populations, further subdivided into RGD and SGD populations). [file 12862_2020_1669_MOESM6_ESM.docx]

**Additional Table S6: Proportion of haplotypes in significant linkage disequilibrium (LD) in chimpanzees (BPRC cohort) and humans (multiple populations, further subdivided into RGD and SGD populations).**

|  |  | Chimpanzees | | |  |  | | |  | Humans | | | |  | |  | | | | | |  |
| --- | --- | --- | --- | --- | --- | --- | --- | --- | --- | --- | --- | --- | --- | --- | --- | --- | --- | --- | --- | --- | --- | --- |
|  |  | BPRC^WB^ | | |  | Multiple populations | | |  | RGD | | | |  | | SGD | | | | | |  |
| Loci pairs |  | *N* | *N_hap_* | *%LD* |  | *k* | $\bar{Nhap} (s.d)$ | $\bar{\%LD} (s.d)$ |  | *k1* | $\bar{Nhap} (s.d)$ | $\bar{\%LD} (s.d)$ | |  | | *k2* | | $\bar{Nhap} (s.d)$ | | | $\bar{\%LD} (s.d)$ |  |
| *DPB1~DQB1* |  | 25 | 18 | 5.56 |  | 40 | 43.15 (22.99) | 15.01 (9.67) |  | 15 | 23.87 (10.15) | | 18.54 (13) | |  | | 25 | | 54.72 (20.65) | 12.9 (6.4) | | |
| *DPB1~DQA1* |  | 25 | 18 | 5.56 |  | 33 | 35.45 (19.69) | 15.63 (11.49) |  | 16 | 21.44 (7.7) | | 18.59 (14.98) | |  | | 17 | | 48.65 (18.39) | 12.86 (6.06) | | |
| *DPB1~DRB1* |  | 25 | 25 | 4 |  | 31 | 53.06 (35.34) | 13.27 (12.34) |  | 16 | 29.75 (12.98) | | 14.82 (16.35) | |  | | 15 | | 77.93 (34.8) | 11.6 (5.88) | | |
| *DPB1~B* |  | 25 | 28 | 3.57 |  | 10 | 72.2 (50.02) | 10.18 (4.46) |  | 4 | 35.5 (13.08) | | 12.72 (4.66) | |  | | 6 | | 96.67 (51.05) | 8.49 (3.76) | | |
| *DPB1~C* |  | 25 | 24 | 4.16 |  | 8 | 60.75 (36.13) | 12.02 (6.49) |  | 2 | 28.5 (23.33) | | 11.94 (5.11) | |  | | 6 | | 71.5 (34.12) | 12.05 (7.33) | | |
| *DPB1~A* |  | 24 | 30 | 6.67 |  | 12 | 52.25 (45.27) | 8.5 (5.03) |  | 5 | 22.6 (8.17) | | 9.44 (7.71) | |  | | 7 | | 73.43 (49.57) | 7.82 (2.35) | | |
| *DQB1~DQA1* |  | 29 | 8 | 12.5 |  | 46 | 20.41 (9.18) | 46.65 (11.68) |  | 20 | 14.2 (8.21) | | 47.97 (13.73) | |  | | 26 | | 25.19 (6.76) | 45.64 (9.99) | | |
| *DQB1~DRB1* |  | 29 | 12 | 16.67 |  | 51 | 12.06 (7.71) | 40.16 (13.10) |  | 23 | 7.61 (4.37) | | 41.07 (15) | |  | | 28 | | 15.82 (6.28) | 39.4 (11.52) | | |
| *DQB1~B* |  | 29 | 23 | 0 |  | 13 | 61.62 (34.97) | 11.68 (6.77) |  | 7 | 38.86 (16.13) | | 14.44 (6.65) | |  | | 6 | | 88.17 (32.42) | 8.46 (5.81) | | |
| *DQB1~C* |  | 29 | 18 | 0 |  | 10 | 51.6 (31.62) | 16.13 (8.36) |  | 5 | 27.6 (15.9) | | 20.23 (10.29) | |  | | 5 | | 75.6 (23.61) | 12.03 (3.05) | | |
| *DQB1~A* |  | 28 | 26 | 0 |  | 16 | 46.56 (33.82) | 8.9 (5.36) |  | 9 | 25.78 (12.88) | | 8.54 (7.26) | |  | | 7 | | 73.29 (34) | 9.37 (1.13) | | |
| *DQA1~DRB1* |  | 29 | 12 | 66.67 |  | 38 | 26.32 (14.3) | 43.51 (11.96) |  | 21 | 18.86 (10.52) | | 44.95 (13.2) | |  | | 17 | | 35.53 (13.09) | 41.73 (10.36) | | |
| *DQA1~B* |  | 29 | 25 | 4 |  | 9 | 54.78 (37.63) | 15.66 (6.75) |  | 5 | 26.25 (14.71) | | 19.8 (5.09) | |  | | 4 | | 87.5 (30.88) | 10.49 (4.78) | | |
| *DQA1~C* |  | 29 | 25 | 0 |  | 6 | 48.17 (28.46) | 18.15 (9.99) |  | 3 | 24 (13.08) | | 21.51 (14.34) | |  | | 3 | | 72.33 (10.07) | 14.8 (3.19) | | |
| *DQA1~A* |  | 28 | 28 | 7.14 |  | 12 | 37.83 (32.3) | 9.78 (6.65) |  | 7 | 17.29 (7.74) | | 10.83 (7.59) | |  | | 5 | | 66.6 (31.71) | 8.31 (5.53) | | |
| *DRB1~B* |  | 29 | 33 | 3.03 |  | 39 | 72.28 (58.11) | 19.27 (10.58) |  | 22 | 42.5 (25.69) | | 24.49 (10.22) | |  | | 17 | | 110.82 (65.99) | 12.51 (6.53) | | |
| *DRB1~C* |  | 29 | 29 | 6.9 |  | 30 | 60.57 (52.64) | 22.57 (10.27) |  | 18 | 30.94 (12.72) | | 27.46 (9.51) | |  | | 12 | | 105 (58.87) | 15.23 (6.35) | | |
| *DRB1~A* |  | 28 | 36 | 2.78 |  | 39 | 59.69 (50.49) | 12.65 (5.82) |  | 24 | 34.88 (23.04) | | 12.64 (6.49) | |  | | 15 | | 99.4 (57.47) | 12.67 (4.8) | | |
| *B~C* |  | 29 | 18 | 27.78 |  | 59 | 54.05 (35.75) | 35.57 (15.62) |  | 21 | 25.67 (21.11) | | 45.9 (18.09) | |  | | 38 | | 69.74 (32.44) | 29.87 (10.57) | | |
| *B~A* |  | 28 | 28 | 17.86 |  | 76 | 90.78 (62.95) | 14.26 (7.87) |  | 27 | 41.22 (37.7) | | 17.78 (9.77) | |  | | 49 | | 118.08 (57.27) | 12.32 (5.86) | | |
| *C~A* |  | 28 | 28 | 17.86 |  | 58 | 67.81 (48.95) | 16.03 (8.23) |  | 23 | 30.35 (25.97) | | 18.67 (10.29) | |  | | 35 | | 92.43 (44.8) | 14.29 (6.11) | | |

*N: number of individuals tested; N_hap_: total number of observed haplotypes; %LD: proportion of observed haplotypes in significant linkage disequilibrium (standardized residual greater than or equal to 1.96 and observed at least 3 times, see Material and Methods); k: number of samples tested,* *k1: number of RGD populations; k2: number of SGD populations;* $\bar{\boldsymbol{N}\boldsymbol{hap}}$*: average number of observed haplotypes;* $\bar{\boldsymbol{\%LD}}\boldsymbol{:}$*average proportion of haplotypes in significant linkage disequilibrium; s.d: standard deviation .*
