# Supplementary material for: Similar patterns of genetic diversity and linkage disequilibrium in Western chimpanzees (Pan troglodytes verus) and humans indicate highly conserved mechanisms of MHC molecular evolution
Source: BMC Evol Biol. 2020 Sep 15;20:119. doi: 10.1186/s12862-020-01669-6 (PMC7491122; doi:10.1186/s12862-020-01669-6)
Supplement: Supplementary file 10 — Additional file 10: Additional Table S10. Nucleotide diversity (П) at Patr loci and other genomic regions in chimpanzees (Pan troglodytes subspecies) and bonobos (Pan paniscus). [file 12862_2020_1669_MOESM10_ESM.docx]

**Additional Table S10: Nucleotide diversity (П) at *Patr* loci and other genomic regions in chimpanzees (Pan troglodytes subspecies) and bonobos (*Pan Paniscus*).**

| Genomic regions | *P.t.verus* | *P.t.troglodytes* | *P.t.schweinfurthii* | *P.t.ellioti* | *Pan Paniscus* |
| --- | --- | --- | --- | --- | --- |
| *Patr-DPB1* | 0.013 | *-* | *-* | *-* | *-* |
| *Patr-DQB1* | 0.039 | *-* | *-* | *-* | *-* |
| *Patr-DQA1* | 0.078 | *-* | *-* | *-* | *-* |
| *Patr-DRB1* | 0.07 | *-* | *-* | *-* | *-* |
| *Patr/Papa-B* | 0.052^1^ | 0.054*^2^* | 0.038^3^ | *-* | 0.037*^2^* |
| *Patr/Papa-C* | 0.021^1^ | 0.025*^2^* | *-* | *-* | 0.014*^2^* |
| *Patr/Papa-A* | 0.027^1^ | 0.032*^2^* | *-* | *-* | 0.024*^2^* |
| *R1*: non-coding autosomal regions^4^ | 0.0008 | 0.002 | 0.0016 | *-* | 0.001 |
| *R2*: non-coding autosomal regions^5^ | 0.0008 | 0.0013 | 0.0009 | *-* | 0.0008 |
| *R3*: Xq13.3^6^ | 0.0005 | 0.0018 | *-* | *-* | *-* |
| *R4*: non-coding autosomal regions^7^ | 0.0008 | 0.0024 | 0.0021 | 0.002 | 0.0009 |
| *R5*: mitogenome^7^ | 0.0069 | 0.0052 | 0.0021 | 0.0027 | 0.0044 |
| *R6*: mitogenome^8^ | 0.0073 | 0.0062 | 0.0027 | 0.0029 | 0.0068 |

*P.t : Pan troglodytes ; -: data not available; the values estimated in this study (Patr genes in P.t. verus) are the values of the pooled cohort of chimpanzees; these data were used in Figure 3.*

*The values for Maibach et al. 2017 have been re-estimated in this study on exon 2 and 3.*

^1^ this study: Individuals are either wild-born or captive-born.

^2^ Maibach et al. 2017: All individuals except one bonobo are from African sanctuaries.

^3^ Wroblewski et al. 2015: All individuals are wild-born and living in natural environment.

^4^ Fischer et al. 2006: All Western and Central chimpanzees are wild-born. Eastern chimpanzees might be born in a Reserve in Kenya. Bonobos are from European zoos.

^5^ Yu et al. 2003: All chimpanzees are either from research laboratories or of unknown origin. Bonobos are from zoos.

^6^ Kaessmann et al. 2001: Eastern chimpanzees are from research laboratories. Western chimpanzees are wild-born or from research laboratories or zoos.

^7^ Fischer et al. 2011: all chimpanzees and bonobos are wild-born except some Western chimpanzees.

^8^ Hvilsom et al. 2014: Chimpanzees and bonobos are either wild-born or born in captivity or of unknown origin.
