# Supplementary material for: Similar patterns of genetic diversity and linkage disequilibrium in Western chimpanzees (Pan troglodytes verus) and humans indicate highly conserved mechanisms of MHC molecular evolution
Source: BMC Evol Biol. 2020 Sep 15;20:119. doi: 10.1186/s12862-020-01669-6 (PMC7491122; doi:10.1186/s12862-020-01669-6)
Supplement: Supplementary file 15 — Additional file 15. Additional Table S15. Percentages of shared allelic frequencies between chimpanzee cohorts at different Patr loci. [file 12862_2020_1669_MOESM15_ESM.docx]

Additional Table S15: Percentages of shared allelic frequencies between chimpanzee cohorts at different *Patr* locus.

| *DPB1* |  | BPRC^wb^ | Texas^cb^ | Yerkes^cb^ | Kuma^wb^ |
| --- | --- | --- | --- | --- | --- |
|  | BPRC^wb^ | 100 |  |  |  |
|  | Texas^cb^ | *-* | - |  |  |
|  | Yerkes^cb^ | *-* | *-* | - |  |
|  | Kuma^wb^ | 72.7 | *-* | *-* | 100 |
| *DQB1* |  | BPRC^wb^ | Texas^cb^ | Yerkes^cb^ | Kuma^wb^ |
|  | BPRC^wb^ | 100 |  |  |  |
|  | Texas^cb^ | 62.5 | 100 |  |  |
|  | Yerkes^cb^ | *-* | *-* | - |  |
|  | Kuma^wb^ | 79.5 | 71.5 | *-* | 100 |
| *DRB1* |  | BPRC^wb^ | Texas^cb^ | Yerkes^cb^ | Kuma^wb^ |
|  | BPRC^wb^ | 100 |  |  |  |
|  | Texas^cb^ | 53.3 | 100 |  |  |
|  | Yerkes^cb^ | *-* | *-* | - |  |
|  | Kuma^wb^ | 65.1 | 63.2 | *-* | 100 |
| *B* |  | BPRC^wb^ | Texas^cb^ | Yerkes^cb^ | Kuma^wb^ |
|  | BPRC^wb^ | 100 |  |  |  |
|  | Texas^cb^ | 60.5 | 100 |  |  |
|  | Yerkes^cb^ | 43.9 | 44.8 | 100 |  |
|  | Kuma^wb^ | *-* | *-* | *-* | - |
| *C* |  | BPRC^wb^ | Texas^cb^ | Yerkes^cb^ | Kuma^wb^ |
|  | BPRC^wb^ | 100 |  |  |  |
|  | Texas^cb^ | *-* | - |  |  |
|  | Yerkes^cb^ | 60.2 | *-* | 100 |  |
|  | Kuma^wb^ | *-* | *-* | *-* | - |
| *A* |  | BPRC^wb^ | Texas^cb^ | Yerkes^cb^ | Kuma^wb^ |
|  | BPRC^wb^ | 100 |  |  |  |
|  | Texas^cb^ | 74.9 | 100 |  |  |
|  | Yerkes^cb^ | 62.6 | 62.2 | 100 |  |
|  | Kuma^wb^ | *-* | *-* | *-* | - |

*Shared allelic frequencies are the complement to 1 of Prevosti’s distances (see Materials and Methods); -: data not available*
