# Supplementary material for: Similar patterns of genetic diversity and linkage disequilibrium in Western chimpanzees (Pan troglodytes verus) and humans indicate highly conserved mechanisms of MHC molecular evolution
Source: BMC Evol Biol. 2020 Sep 15;20:119. doi: 10.1186/s12862-020-01669-6 (PMC7491122; doi:10.1186/s12862-020-01669-6)
Supplement: Supplementary file 16 — Additional file 16: Additional Table S15. Percentages of shared allelic frequencies between chimpanzee cohorts at different Patr locus. [file 12862_2020_1669_MOESM16_ESM.docx]

**Vangenot et al.**

**Similar patterns of genetic diversity and linkage disequilibrium in Western chimpanzees (Pan troglodytes verus) and humans indicate highly conserved mechanisms of MHC molecular evolution**

**1. Supplementary Material on methods**

1.1. Description and recoding of chimpanzees and human population samples

1.2. Linkage disequilibrium

**2. Supplementary Material on results**

2.1. Allele frequencies in chimpanzees and humans

2.2. MHC genetic diversity in wild-born and captive-born Western chimpanzee cohorts

**3. References**

**1. Supplementary Material on methods**

**1.1. Description and recoding of chimpanzees and human population samples**

Multi-locus MHC genotypes of chimpanzee cohorts and human population samples (including loci A, B, C, DRB1, DQA1, DQB1 and/or DPB1) are of alleles defined at the 2^nd^ field level of resolution according to the official nomenclatures of the IPD-MHC. All alleles were first translated into DNA sequences (of exon 2 and 3, for class I, and of exon 2, for class II alleles) by using the IPD/MHC and IPD/IMGT-HLA resources [1, 2] (<http://www.ebi.ac.uk/ipd/mhc/nhp/>, downloaded in May 2013 for loci A, B, C and DRB1 and August 2018 for loci DQA1, DQB1 and DPB1).

In order to analyze data, MHC genotypes of chimpanzee cohorts and human population samples were first recoded into the file format UNIFORMAT where data with ambiguities can be represented and analysed. Ambiguities occur when, depending on the typing method used, multiple possible genotypes may exist for the same individual and/or when phenotypes exhibiting a single allele can either be interpreted as homozygous or heterozygous with an unknown (e.g. yet undiscovered) allele. In the last case, a “blank” allele is used to represent all alleles that are not observable in the phenotype [3]. Genotypes of each individual of the chimpanzees’ cohorts are in **Additional table S13** and the number of individuals to each genotype is presented in **Additional Table S14.**

**1.2. Global Linkage disequilibrium**

We assessed global linkage disequilibrium using a non-parametric procedure to produce an empirical distribution for a linkage disequilibrium statistic (LRT, defined below) generated through a resampling procedure.

A Likelihood Ratio Test (LRT) is used to compare two estimates: the log likelihood of the estimated haplotype frequency and the sum of the log likelihoods of the allele frequencies estimates. Using two-locus genotypes, the test estimates, on the one hand, the frequency distribution of haplotypes (defined by the combination of two alleles at the two loci) and, on the other hand, the frequency distribution of alleles at each locus. The log likelihood of the distribution of haplotypes is then compared to the sum of the log likelihoods of the frequency distributions of alleles at each locus. Under the null hypothesis of linkage equilibrium, we expect those two statistics to have the same value.

Since the approximation by a chi-square distribution is not applicable in case of small sample sizes with high number of alleles, this LRT test is complemented with a resampling procedure (PRS), where the null hypothesis of linkage equilibrium is used to generate a given number of two-locus samples, by bootstrapping the allele frequency estimates, to which the LRT test is applied. The procedure provides an empirical distribution for the LRT statistic under the hypothesis of linkage equilibrium, and the reported *P*-value is the position (quantile) of the observed LRT in this empirical distribution.

The input of the Likelihood Ratio Test (LRT) algorithm is the sample of the observed genotypes (unphased haplotypes), from which haplotypes and allele frequencies are estimated by an EM algorithm.

To assess the proportion of haplotypes in linkage disequilibrium, we used a standardized measure of the contribution of each putative haplotype to a χ2 test (named « standardized residuals »). As this standardized residuals are expected to follow a Normal distribution, haplotypes were considered in significant linkage disequilibrium when the standardized residuals were greater than 1.96 or lower than -1.96 and observed more than 3 times.

**1.3. Simulations**

In order to control for the great discrepancy in sample size between chimpanzee cohorts and human populations we performed some simulations through a resampling procedure.

For each human population sample and each locus, we randomly draw 1,000 samples of the same size as the pooled cohort of chimpanzees (i.e. N=44 for DPB1, 48 for DQB1, 29 for DQA1, 46 for DRB1, 51 for B, 51 for C and 50 for A). We thus obtained 491,000 simulated samples.

On these simulated samples, we tested Hardy-Weinberg equilibrium, estimated the 3 diversity indices, heterozygosity, nucleotide diversity and allelic richness and we applied the Ewens-Watterson selective neutrality test. In addition, we assessed global linkage disequilibrium and estimated the proportion of haplotypes in significant linkage disequilibrium in simulated samples for pairs *DQB1~DRB1(N=46), DQB1~B (N=29), DQA1~DRB1 (N=29), DQB1~DQA1 (N=29), DQA1~DQB1 (N=29), DRB1~B (N=29), DRB1~C (N=29), B~C (N=51), B~A (N=50), C~A (N=50)*.

**2. Supplementary Material on results**

**2.1. Allele frequencies in chimpanzees and humans**

Although many alleles are common to all cohorts, their frequencies sometimes differ substantially between them, as indicated by some relatively low percentages of shared frequencies, e.g. 44-45% between Yerkes^cb^ and both BRPC^wb^ and Texas^cb^ at locus *B*, and 53% between BRPC^wb^ and Texas^cb^ at locus *DRB1* (**Additional Table S15**).

Direct comparisons of allele frequencies between chimpanzees and humans were not possible because no identical alleles are found in the two species. However, phylogenetic analyses have shown that MHC lineages are shared between the two species [4-6], in agreement with a trans-species mode of evolution [7-9]. We thus mainly compared MHC lineages.

At locus *A*, all *Patr-A* alleles group with the HLA-A1/A3/A11/A30 family (A3 lineage) [4, 6], which represents a small proportion of *HLA*-*A* frequencies (24.2%±12.3%) in human populations [10].

The phylogenetic tree of locus *B* has low structure due to the frequent exchange of polymorphic segments between alleles [6], which complicates inter-species comparison. However, comparisons have been done at the peptide-binding level and have shown that three *Patr-B* alleles, *B*01:01*, *B*03:01* and *B*05:01*, share similar peptide binding motifs with *HLA-B*27:05* and *B*57:01* [11], these two alleles being strongly associated with resistance to the development of AIDS in humans. We have found that the three *Patr* alleles *B*01:01*, *B*03:01* and *B*05:01* reached a cumulated frequency of 50% in BPRC^wb^ and Texas^cb^ and 41% in Yerkes^cb^, and the two HLA alleles *B*27:05* and *B*57:01* a cumulated frequency from 0 to 11.5% in human populations.

Two of the three most frequent *Patr-C* alleles found in this study, *C*04:01* and C*09:01 (cumulated frequency of 43%), segregate with alleles of the HLA-C*07 lineage [4, 6]. The latter reach an average cumulated frequency of 21% in the studied human populations and up to 40% in European populations [12].

Some *DRB* lineages are old entities and are suggested to date back before the divergence of Old World Monkeys and hominids such as *DRB6/DRB2* and *DRB5* [13]. For DRB, humans share the lineage *HLA-DRB1*15* with the chimpanzee lineage *Patr-DRB1*02* according to analyses of exon 2, *HLA-DRB1*07/09* with the chimpanzee lineage *Patr-DRB1*07*, *HLA/Patr-DRB1*10* and no *Patr* alleles segregate within the *HLA-DRB1*04* lineage [13-15]. In this study, in the pooled cohort of chimpanzees, *Patr-DRB1*02* is observed at a frequency of more than 40% and both *Patr-DRB1*07* and -*DRB1*10* at a frequency of 6%. In humans, *HLA-DRB1*15*, *-DRB1*07/09* and *-DRB1*10* are observed at frequencies ranging up to 52%, up to 25% and up to 15%, respectively.

At the *DQA1* locus, 50% of *Patr* allele are from the *DQA1*01* and *DQA1*05* lineages shared with humans [16, 17]. The *HLA* alleles of these two lineages are among the most frequent in human populations (average frequencies of 33% and 27%, respectively).

At *DQB1*, 95% of allele frequencies observed in chimpanzees are from the *DQB1*03* and *DQB1*06* lineages shared with both Cercopithecoidea and humans, their frequencies in humans reaching a cumulated frequency of about 58%.

By comparing the frequencies of MHC lineage shared between Western chimpanzees and humans, we thus note a main difference at locus *A* (100% of *Patr* alleles correspond to only 24% of *HLA* alleles), the other loci showing similar frequency patterns for many lineages.

**2.2. MHC genetic diversity in wild-born and captive-born Western chimpanzee cohorts**

Despite a similar pattern of diversity at different *Patr* loci in the four Western chimpanzee cohorts, we found that the two captive-born cohorts, Texas^cb^ and Yerkes^cb^, display higher heterozygosity and allelic richness than the two wild-born cohorts, BPRC^wb^ and Kuma^wb^, at all loci where data are available (*Patr-DQB1*, -*DRB1*, -*B*, -*C*, and -*A*). Differences in nucleotide diversity between these two types of cohorts are less marked but show the same tendency. This result is surprising because captive animals are generally described as having a lower diversity, due to lower effective population size and inbreeding. Moreover, the study of [18] did not observe any difference of diversity between wild and captive chimpanzees. However, as there is no information on how many generations in captivity the captive-born chimpanzees come from, if the captive-born chimpanzees are the offspring of two wild-born chimpanzees one may not expect a difference between them and the wild-born chimpanzees. In addition, there is uncertainty about the origin of individuals in the captive-born cohorts that we studied: whereas both the place of origin and the sub-species (*P.t.verus*) were known for all BPRC^wb^ and Kuma^wb^ chimpanzees, this was not the case for Texas^cb^ [19]. We also noticed that two alleles found in Texas^cb^, *B*23:04* and *B*23:02*, were observed among Eastern (*P.t.schweinfurthii*) and Central (*P.t.troglodytes*) chimpanzees, respectively [4, 20-22], despite the fact that no *Patr-B* alleles are supposed to be shared between different sub-species of chimpanzees [21]. As these two alleles appear with alleles commonly found in *P.t.verus* in the corresponding genotypes, this strongly suggests the presence of at least two hybrids in Texas^cb^, one *P.t.verus / P.t.schweinfurthii* and one *P.t.verus / P.t.troglodytes,* likely inflating the allelic richness and the genetic diversity in this captive-born cohort. Also, the determination as *P.t.verus* of the other captive-born cohort, Yerkes^cb^, was based exclusively on mitochondrial DNA and pedigree analyses [23], which can be misleading especially in the presence of hybrids [24]. Nine alleles were observed uniquely among Yerkes^cb^ individuals, two of which, *B*22:01* and *B*23:01*, were observed among Central (*P.t.troglodytes*) chimpanzees [22]. Although we cannot exclude a sampling effect, these alleles are likely to come from other sub-species, for example *P.t.troglodytes* and *P.t.ellioti* (Nigeria/Cameroon) for which very few animals have been analysed to date and whose allelic repertoire is largely unknown. This is supported by the Prevosti distances that we calculated between the cohorts, indicating that Yerkes^cb^ is more distant from Texas^cb^ and BPRC^wb^ than these latter two are from each other (**Additional Table S15**). Therefore, our results may be explained by the presence of individuals from other sub-species or hybrids in the captive-born cohorts. These observations call for a better determination of sub-species, especially if animals are studied from a biomedical perspective considering that the different sub-species of chimpanzees are susceptible to different pathogens [11] or destined to reintroduction in the wild.

**3. References**

1. Maccari G, Robinson J, Ballingall K, Guethlein LA, Grimholt U, Kaufman J, Ho CS, de Groot NG, Flicek P, Bontrop RE *et al*: **IPD-MHC 2.0: an improved inter-species database for the study of the major histocompatibility complex**. *Nucleic Acids Res* 2017, **45**(D1):D860-d864.

2. de Groot NG, Otting N, Maccari G, Robinson J, Hammond JA, Blancher A, Lafont BAP, Guethlein LA, Wroblewski EE, Marsh SGE *et al*: **Nomenclature report 2019: major histocompatibility complex genes and alleles of Great and Small Ape and Old and New World monkey species**. *Immunogenetics* 2019.

3. Nunes JM, Buhler S, Sanchez-Mazas A: **NO to obsolete definitions: YES to blanks**. *Tissue Antigens* 2014, **83**(2):119-120.

4. de Groot NG, Otting N, Argüello R, Watkins DI, Doxiadis GG, Madrigal JA, Bontrop RE: **Major histocompatibility complex class I diversity in a West African chimpanzee population: implications for HIV research**. *Immunogenetics* 2000, **51**(6):398-409.

5. McAdam SN, Boyson JE, Liu X, Garber TL, Hughes AL, Bontrop RE, Watkins DI: **Chimpanzee MHC class I A locus alleles are related to only one of the six families of human A locus alleles**. *Journal of immunology (Baltimore, Md : 1950)* 1995, **154**(12):6421-6429.

6. Adams EJ, Cooper S, Thomson G, Parham P, Adams E: **Common chimpanzees have greater diversity than humans at two of the three highly polymorphic MHC class I genes**. *Immunogenetics* 2000, **51**(6):410-424.

7. Mayer WE, Jonker M, Klein D, Ivanyi P, van Seventer G, Klein J: **Nucleotide sequences of chimpanzee MHC class I alleles: evidence for trans-species mode of evolution**. *EMBO J* 1988, **7**(9):2765-2774.

8. Figueroa F, Gunther E, Klein J: **MHC polymorphism pre-dating speciation**. *Nature* 1988, **335**(6187):265-267.

9. Klein J, Sato A, Nikolaidis N: **MHC, TSP, and the origin of species: from immunogenetics to evolutionary genetics**. *Annu Rev Genet* 2007, **41**:281-304.

10. Buhler S, Sanchez-Mazas A: **HLA DNA sequence variation among human populations: molecular signatures of demographic and selective events**. *PloS one* 2011, **6**(2):e14643.

11. de Groot NG, Bontrop RE: **The HIV-1 pandemic: does the selective sweep in chimpanzees mirror humankind's future?** *Retrovirology* 2013, **10**:53.

12. Nunes JM, Buhler S, Roessli D, Sanchez-Mazas A, collaboration H-n: **The HLA-net GENE[RATE] pipeline for effective HLA data analysis and its application to 145 population samples from Europe and neighbouring areas**. *Tissue Antigens* 2014, **83**(5):307-323.

13. Doxiadis GG, Hoof I, de Groot N, Bontrop RE: **Evolution of HLA-DRB genes**. *Molecular biology and evolution* 2012, **29**(12):3843-3853.

14. Doxiadis GG, de Groot N, de Groot NG, Doxiadis II, Bontrop RE: **Reshuffling of ancient peptide binding motifs between HLA-DRB multigene family members: old wine served in new skins**. *Mol Immunol* 2008, **45**(10):2743-2751.

15. de Groot NG, Heijmans CM, de Groot N, Doxiadis GG, Otting N, Bontrop RE: **The chimpanzee Mhc-DRB region revisited: gene content, polymorphism, pseudogenes, and transcripts**. *Mol Immunol* 2009, **47**(2-3):381-389.

16. Bontrop RE, Otting N, de Groot NG, Doxiadis GG: **Major histocompatibility complex class II polymorphisms in primates**. *Immunol Rev* 1999, **167**:339-350.

17. Otting N, de Groot NG, Doxiadis GG, Bontrop RE: **Extensive Mhc-DQB variation in humans and non-human primate species**. *Immunogenetics* 2002, **54**(4):230-239.

18. Hvilsom C, Frandsen P, Borsting C, Carlsen F, Salle B, Simonsen BT, Siegismund HR: **Understanding geographic origins and history of admixture among chimpanzees in European zoos, with implications for future breeding programmes**. *Heredity (Edinb)* 2013, **110**(6):586-593.

19. Caggiari L, Simula MP, Marzotto A, Shiina M, Rehermann B, De Re V: **Identification of novel chimpanzee MHC class I and II alleles using an improved sequence-based typing strategy**. *Hum Immunol* 2006, **67**(1-2):63-72.

20. Cooper S, Adams EJ, Wells RS, Walker CM, Parham P: **A major histocompatibility complex class I allele shared by two species of chimpanzee**. *Immunogenetics* 1998, **47**(3):212-217.

21. Wroblewski EE, Norman PJ, Guethlein LA, Rudicell RS, Ramirez MA, Li Y, Hahn BH, Pusey AE, Parham P: **Signature Patterns of MHC Diversity in Three Gombe Communities of Wild Chimpanzees Reflect Fitness in Reproduction and Immune Defense against SIVcpz**. *PLoS Biology* 2015, **13**(5):e1002144.

22. Maibach V, Hans JB, Hvilsom C, Marques-Bonet T, Vigilant L: **MHC class I diversity in chimpanzees and bonobos**. *Immunogenetics* 2017.

23. Adams EJ, Cooper S, Parham P: **A novel, nonclassical MHC class I molecule specific to the common chimpanzee**. *Journal of Immunology* 2001, **167**(7):3858-3869.

24. Becquet C, Patterson N, Stone AC, Przeworski M, Reich D: **Genetic structure of chimpanzee populations**. *PLoS Genet* 2007, **3**(4):e66.
