# Supplementary material for: Similar patterns of genetic diversity and linkage disequilibrium in Western chimpanzees (Pan troglodytes verus) and humans indicate highly conserved mechanisms of MHC molecular evolution
Source: BMC Evol Biol. 2020 Sep 15;20:119. doi: 10.1186/s12862-020-01669-6 (PMC7491122; doi:10.1186/s12862-020-01669-6)
Supplement: Supplementary file 17 — Additional file 17: Additional Figure S1. Genetic diversity in chimpanzees (pooled cohort) and humans (multiple populations). A) Allelic richness B) heterozygosity, C) nucleotide diversity at each locus under study for the pooled cohort of chimpanzees (in red empty circle) and for the human populations (in blue) represented as violin plots; An average number of k = 70 (s.d 15.9) human population samples of average size N = 109.2 (s.d 17.31) were used. The width of the violin varies so as to represent the probability density of the data, the thick blue bar in the centre represents the interquartile range, the thin black line extended from it represents the 95% confidence intervals, and the green dot is the median. The MHC loci are presented according to their position on the chromosome from the centromere (left) to the telomere (right). [file 12862_2020_1669_MOESM17_ESM.pdf]

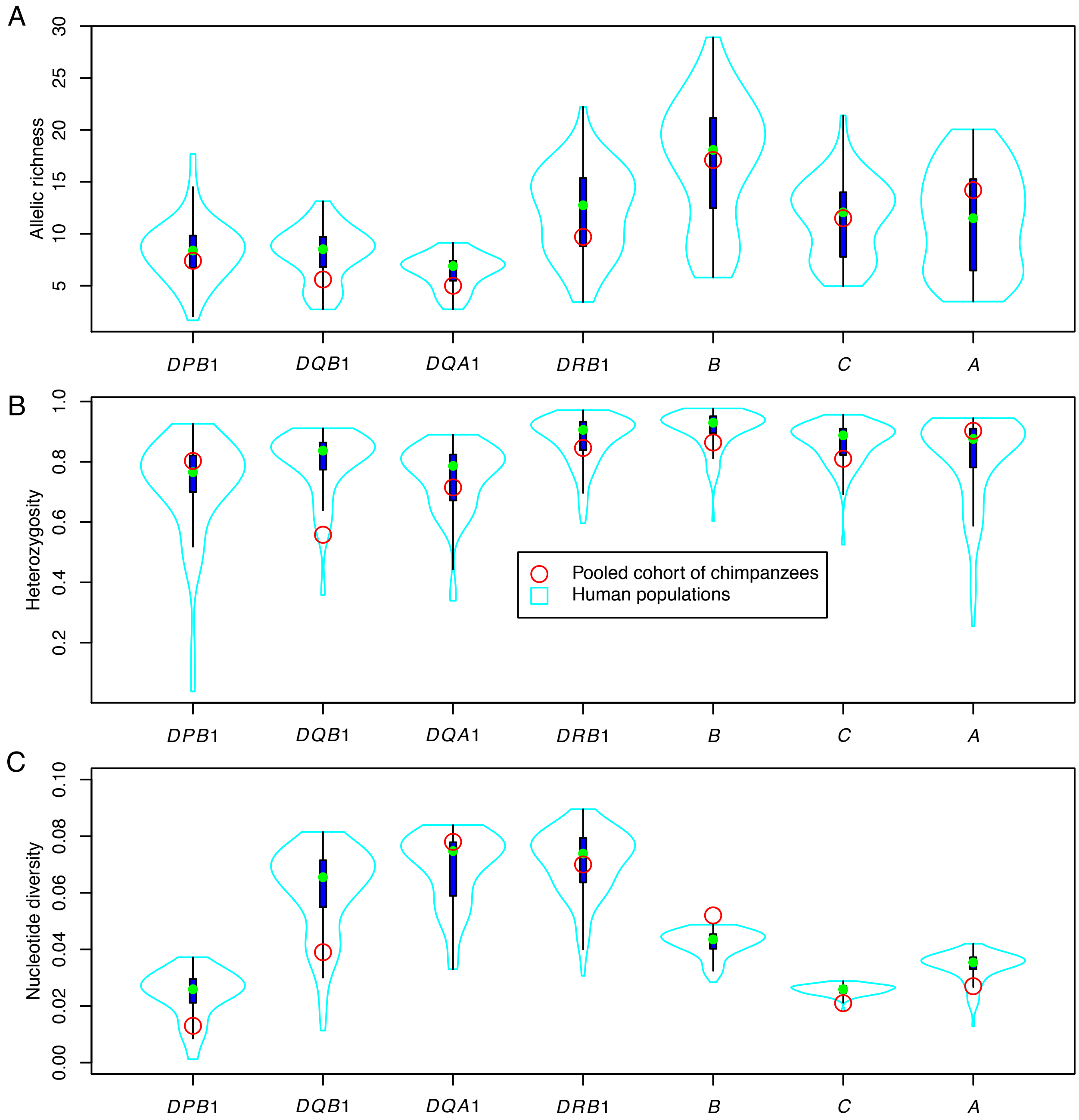

A
Allelic richness
30
25
20
15
10
5
DPB1
DQB1
DQA1
DRB1
B
C
A
B
Heterozygosity
1.0
0.8
0.6
0.4
0.2
Pooled cohort of chimpanzees
Human populations
C
Nucleotide diversity
0.10
0.08
0.06
0.04
0.02
0.00

Additional Figure S1: Genetic diversity in chimpanzees (pooled cohort) and humans (multiple populations). A) Allelic richness B) heterozygosity, C) nucleotide diversity at each locus under study for the pooled cohort of chimpanzees (in red empty circle) and for the human populations (in blue) represented as violin plots; An average number of k=70 (s.d 15.9) human population samples of average size N=109.2 (s.d 17.31) were used. The width of the violin varies so as to represent the probability density of the data, the thick blue bar in the centre represents the interquartile range, the thin black line extended from it represents the 95% confidence intervals, and the green dot is the median. The MHC loci are presented according to their position on the chromosome from the centromere (left) to the telomere (right).
