# Supplementary material for: Similar patterns of genetic diversity and linkage disequilibrium in Western chimpanzees (Pan troglodytes verus) and humans indicate highly conserved mechanisms of MHC molecular evolution
Source: BMC Evol Biol. 2020 Sep 15;20:119. doi: 10.1186/s12862-020-01669-6 (PMC7491122; doi:10.1186/s12862-020-01669-6)
Supplement: Supplementary file 18 — Additional file 18: Additional Figure S2. Genetic diversity in chimpanzees (pooled cohort) and humans (multiple populations and simulated populations). A) Allelic richness B) heterozygosity, C) nucleotide diversity at each locus under study for the pooled cohort of chimpanzees (in black filled circle) and for the human populations represented as violin plots. Diversity values of each human population sample are plotted as a violin plot in red; Simulated diversity values (1000 samples of the same size per locus as the pooled cohort of chimpanzees) are plotted as a violin plot in blue. The width of the violin varies so as to represent the probability density of the data, the thick blue bar in the centre represents the interquartile range, the thin black line extended from it represents the 95% confidence intervals, and the green dot is the median. The MHC loci are presented according to their position on the chromosome from the centromere (left) to the telomere (right). [file 12862_2020_1669_MOESM18_ESM.pdf]

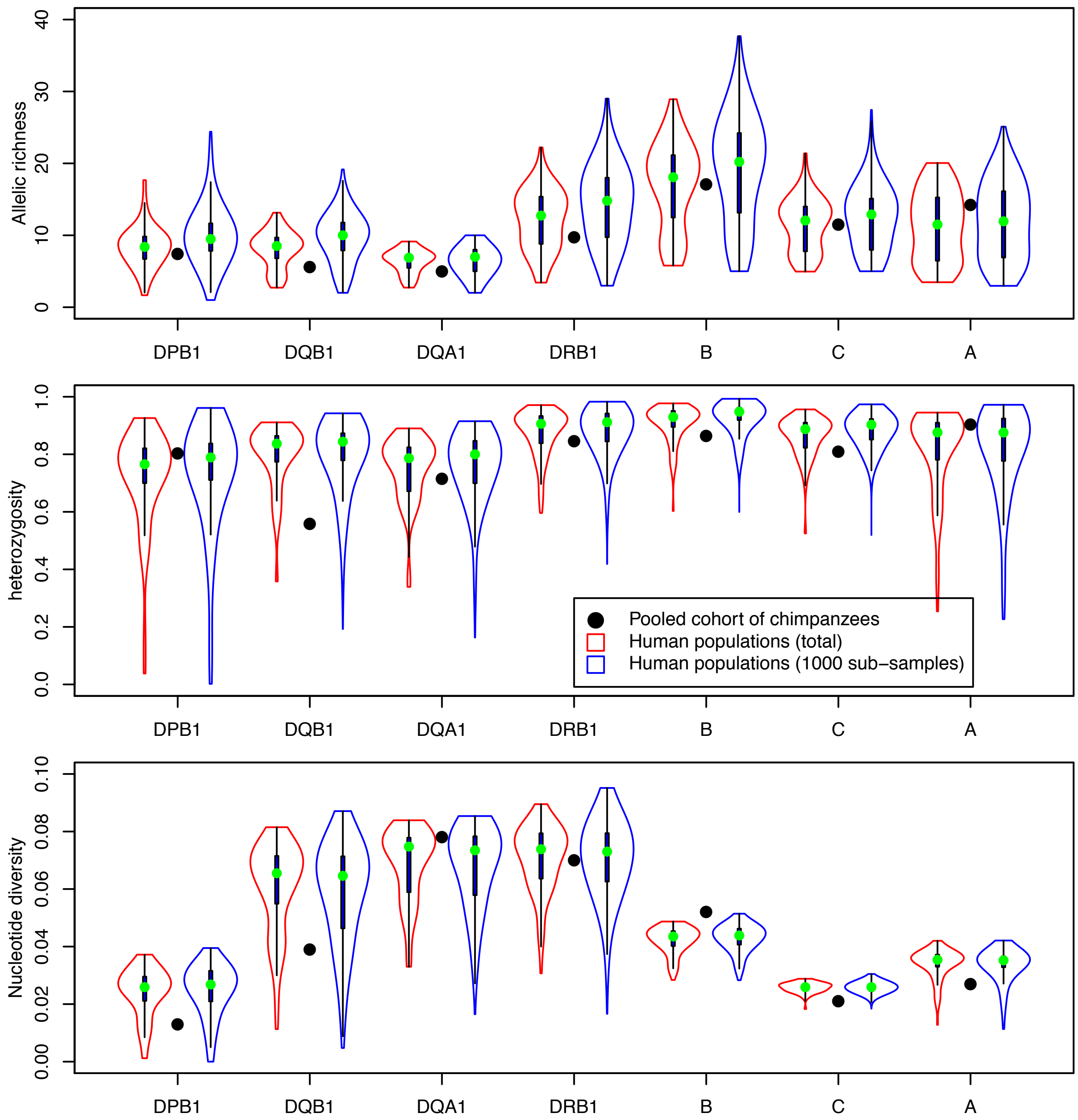

Allelic richness
0
10
20
30
40
DPB1
DQB1
DQA1
DRB1
B
C
A
heterozygosity
0.0
0.2
0.4
0.6
0.8
1.0
Pooled cohort of chimpanzees
Human populations (total)
Human populations (1000 sub−samples)
Nucleotide diversity
0.00
0.02
0.04
0.06
0.08
0.10

Additional Figure S2: Genetic diversity in chimpanzees (pooled cohort) and humans (multiple populations and simulated populations). A) Allelic richness B) heterozygosity, C) nucleotide diversity at each locus under study for the pooled cohort of chimpanzees (in black filled circle) and for the human populations represented as violin plots. Diversity values of each human population sample are plotted as a violin plot in red; Simulated diversity values (1000 samples of the same size per locus as the pooled cohort of chimpanzees) are plotted as a violin plot in blue. The width of the violin varies so as to represent the probability density of the data, the thick blue bar in the centre represents the interquartile range, the thin black line extended from it represents the 95% confidence intervals, and the green dot is the median. The MHC loci are presented according to their position on the chromosome from the centromere (left) to the telomere (right).
