# Supplementary material for: Similar patterns of genetic diversity and linkage disequilibrium in Western chimpanzees (Pan troglodytes verus) and humans indicate highly conserved mechanisms of MHC molecular evolution
Source: BMC Evol Biol. 2020 Sep 15;20:119. doi: 10.1186/s12862-020-01669-6 (PMC7491122; doi:10.1186/s12862-020-01669-6)
Supplement: Supplementary file 19 — Additional file 19: Additional Figure S3. Genetic diversity in chimpanzees (multiple cohorts) and humans (multiple populations) averaged by geographic regions. A) Allelic richness B) heterozygosity, C) nucleotide diversity at each locus under study for each cohort of chimpanzees and for the human populations where samples are grouped by geographic regions; The values calculated for each chimpanzee cohort are indicated by filled and unfilled shapes in red for cohorts of wild-born and captive-born chimpanzees, respectively; for the human populations, each region is represented by a triangle of different colour as defined in legend. The MHC loci are presented according to their position on the chromosome from the centromere (left) to the telomere (right). [file 12862_2020_1669_MOESM19_ESM.pdf]

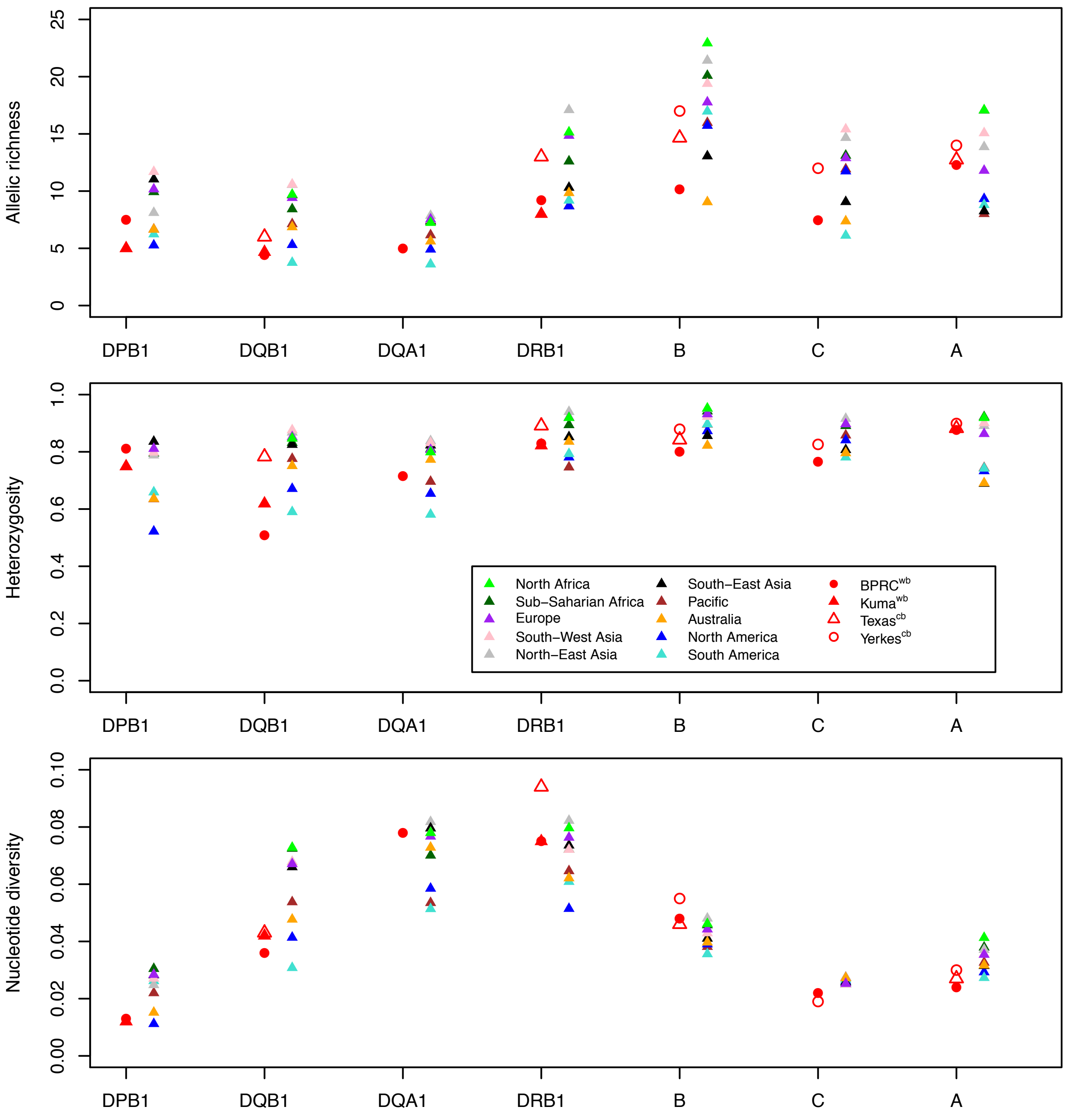

Allelic richness
Heterozygosity
Nucleotide diversity
DPB1
DQB1
DQA1
DRB1
B
C
A
North Africa
Sub−Saharian Africa
Europe
South−West Asia
North−East Asia
South−East Asia
Pacific
Australia
North America
South America
BPRC^wb
Kuma^wb
Texas^cb
Yerkes^cb

Additional Figure S3: Genetic diversity in chimpanzees (multiple cohorts) and humans (multiple populations) averaged by geographic regions. A) Allelic richness B) heterozygosity, C) nucleotide diversity at each locus under study for each cohort of chimpanzees and for the human populations where samples are grouped by geographic regions; The values calculated for each chimpanzee cohort are indicated by filled and unfilled shapes in red for cohorts of wild-born and captive-born chimpanzees, respectively; for the human populations, each region is represented by a triangle of different colour as defined in legend. The MHC loci are presented according to their position on the chromosome from the centromere (left) to the telomere (right).
