# Supplementary material for: Similar patterns of genetic diversity and linkage disequilibrium in Western chimpanzees (Pan troglodytes verus) and humans indicate highly conserved mechanisms of MHC molecular evolution
Source: BMC Evol Biol. 2020 Sep 15;20:119. doi: 10.1186/s12862-020-01669-6 (PMC7491122; doi:10.1186/s12862-020-01669-6)
Supplement: Supplementary file 20 — Additional file 20: Additional Figure S4. Allele frequency distributions for class I and class II loci in the cohorts of chimpanzees including the pooled cohort. 1: locus B, 2: locus C, 3: locus A, 4: locus DPB1, 5: locus DQB1, 6: locus DQA1, and 7: locus DRB1. Alleles are represented by different colours as defined in the legend. Colours in the legend follow the same order as allele frequencies in the plot. Values are in Additional Table S3. [file 12862_2020_1669_MOESM20_ESM.pdf]

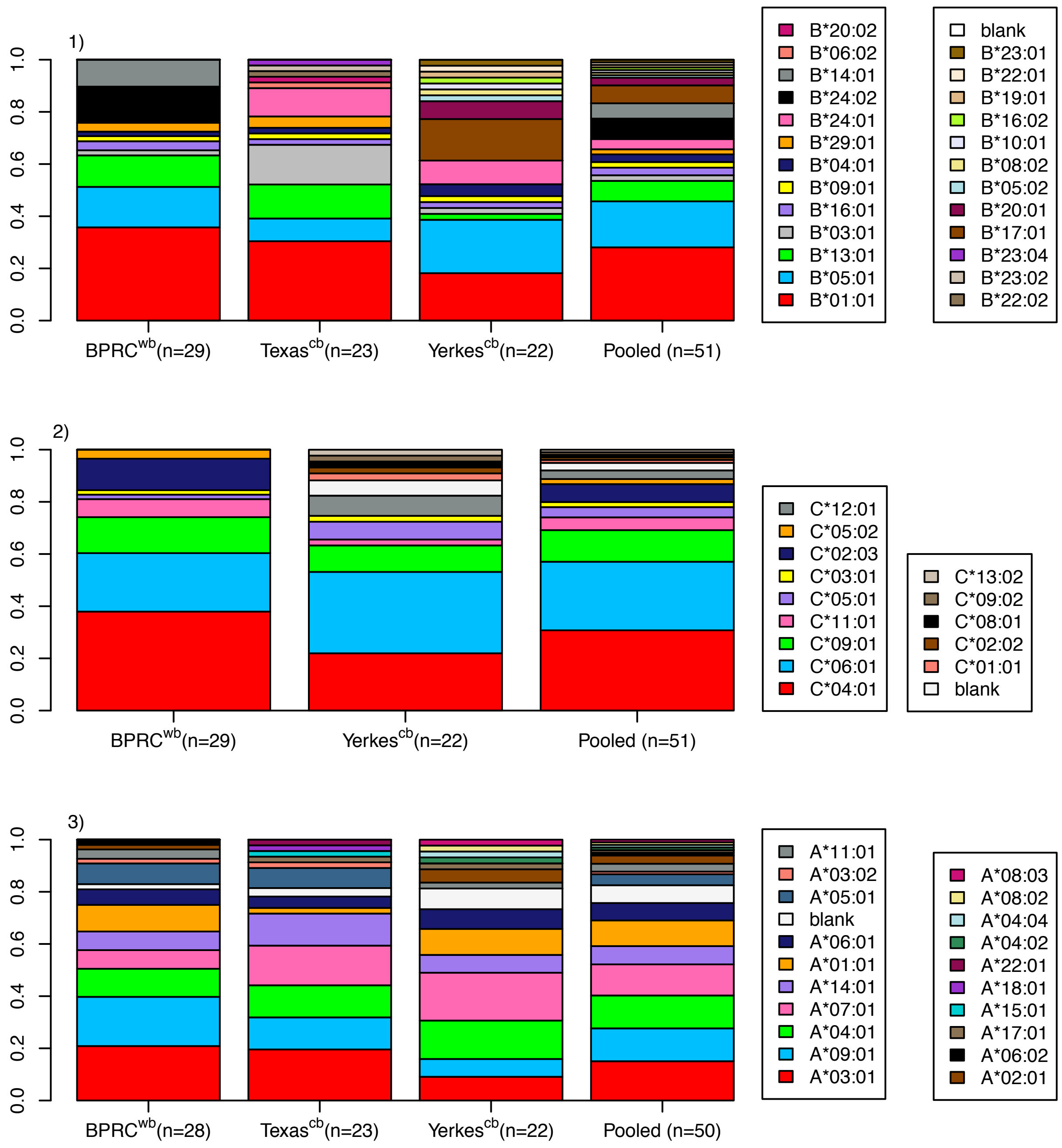
1)
1.0
0.8
0.6
0.4
0.2
0.0
BPRC^wb(n=29)
Texas^cb(n=23)
Yerkes^cb(n=22)
Pooled (n=51)
B*20:02
B*06:02
B*14:01
B*24:02
B*24:01
B*29:01
B*04:01
B*09:01
B*16:01
B*03:01
B*13:01
B*05:01
B*01:01
blank
B*23:01
B*22:01
B*19:01
B*16:02
B*10:01
B*08:02
B*05:02
B*20:01
B*17:01
B*23:04
B*23:02
B*22:02
2)
BPRC^wb(n=29)
Yerkes^cb(n=22)
Pooled (n=51)
C*12:01
C*05:02
C*02:03
C*03:01
C*05:01
C*11:01
C*09:01
C*06:01
C*04:01
C*13:02
C*09:02
C*08:01
C*02:02
C*01:01
blank
3)
BPRC^wb(n=28)
Texas^cb(n=23)
Yerkes^cb(n=22)
Pooled (n=50)
A*11:01
A*03:02
A*05:01
blank
A*06:01
A*01:01
A*14:01
A*07:01
A*04:01
A*09:01
A*03:01
A*08:03
A*08:02
A*04:04
A*04:02
A*22:01
A*18:01
A*15:01
A*17:01
A*06:02
A*02:01

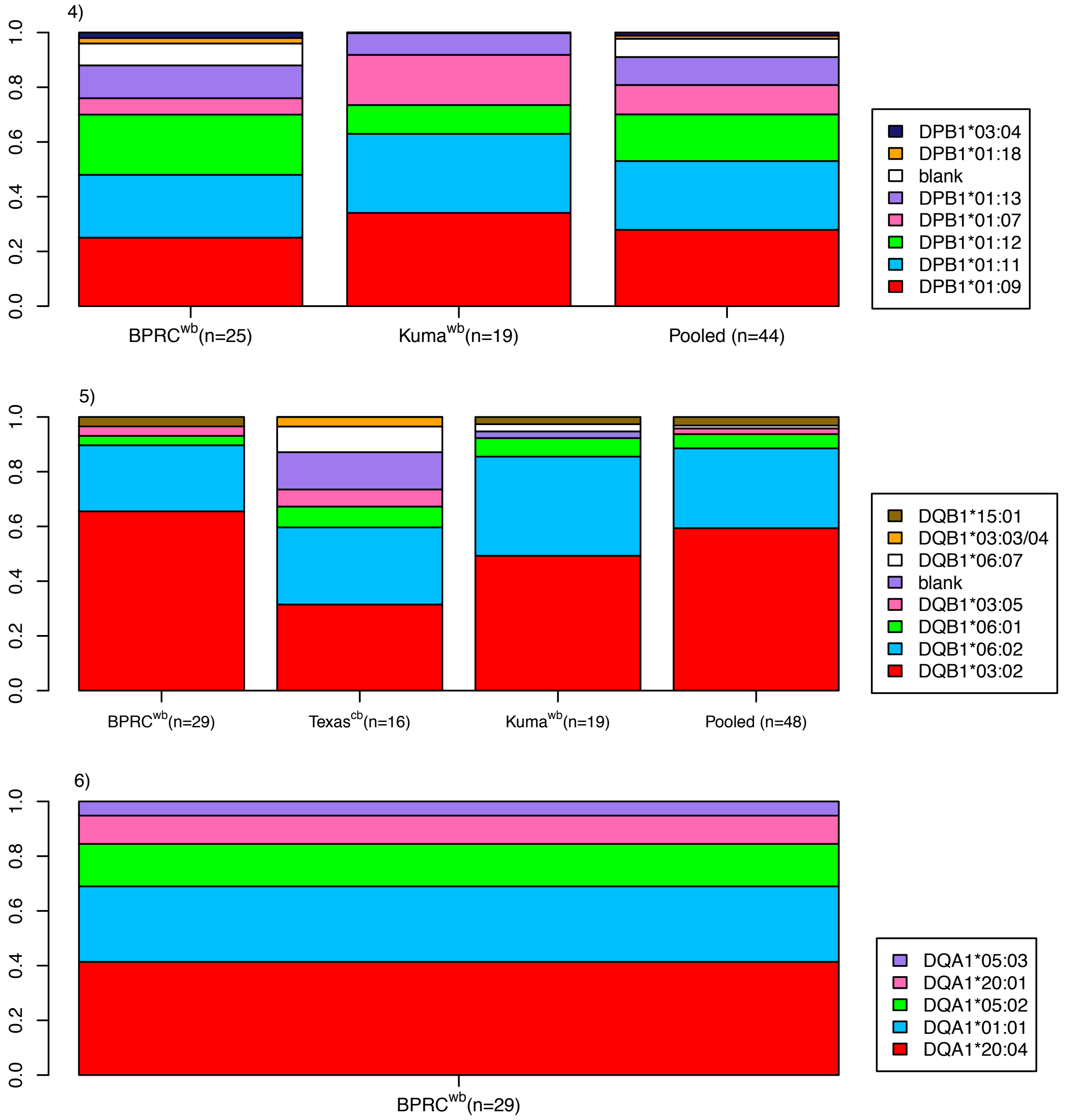
4)
1.0
0.8
0.6
0.4
0.2
0.0
BPRC^{wb}(n=25)
Kuma^{wb}(n=19)
Pooled (n=44)
DPB1*03:04
DPB1*01:18
blank
DPB1*01:13
DPB1*01:07
DPB1*01:12
DPB1*01:11
DPB1*01:09
5)
BPRC^{wb}(n=29)
Texas^{cb}(n=16)
Kuma^{wb}(n=19)
Pooled (n=48)
DQB1*15:01
DQB1*03:03/04
DQB1*06:07
blank
DQB1*03:05
DQB1*06:01
DQB1*06:02
DQB1*03:02
6)
BPRC^{wb}(n=29)
DQA1*05:03
DQA1*20:01
DQA1*05:02
DQA1*01:01
DQA1*20:04

7)

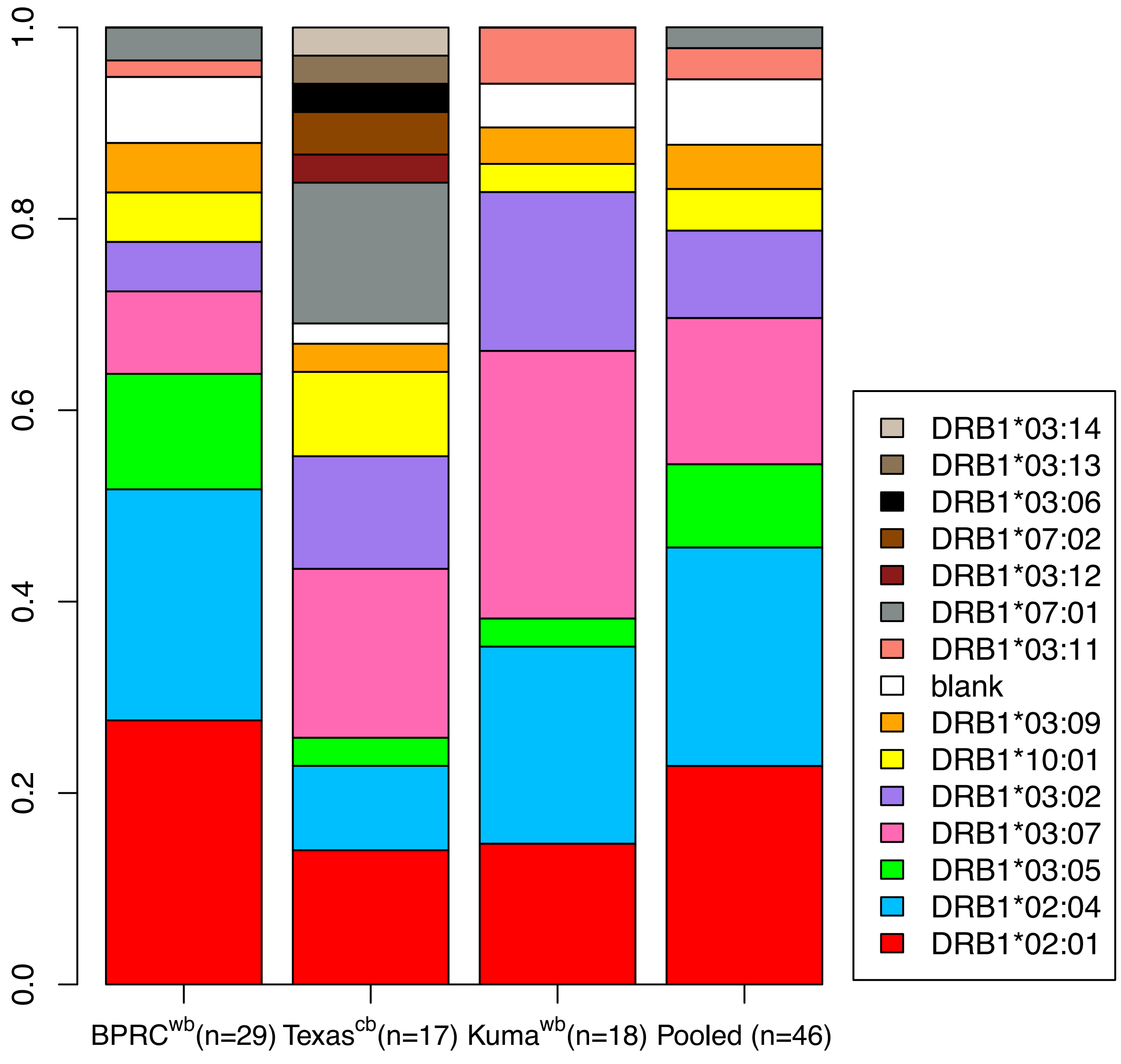

0.0
0.2
0.4
0.6
0.8
1.0
BPRCwb(n=29)
Texascb(n=17)
Kumawb(n=18)
Pooled (n=46)
DRB1*03:14
DRB1*03:13
DRB1*03:06
DRB1*07:02
DRB1*03:12
DRB1*07:01
DRB1*03:11
blank
DRB1*03:09
DRB1*10:01
DRB1*03:02
DRB1*03:07
DRB1*03:05
DRB1*02:04
DRB1*02:01

Additional Figure S4: Allele frequency distributions for class I and class II loci in the cohorts of chimpanzees including the pooled cohort. 1: locus B, 2: locus C, 3: locus A, 4: locus DPB1, 5: locus DQB1, 6: locus DQA1, and 7: locus DRB1. Alleles are represented by different colours as defined in the legend. Colours in the legend follow the same order as allele frequencies in the plot. Values are in Additional Table S3.
